# Shifting gears: Study of immune system parameters of male habitual marathon runners

**DOI:** 10.3389/fimmu.2022.1009065

**Published:** 2023-01-13

**Authors:** Ioannis Panagoulias, Nikolaos Charokopos, Iason Thomas, Panagiota I. Spantidea, Anne-Lise de Lastic, Maria Rodi, Spyridoula Anastasopoulou, Ioanna Aggeletopoulou, Charalampos Lazaris, Kiriakos Karkoulias, Lydia Leonidou, Neoklis A. Georgopoulos, Kostas B. Markou, Athanasia Mouzaki

**Affiliations:** ^1^ Laboratory of Immunohematology, Division of Hematology, Department of Internal Medicine, Medical School, University of Patras, Patras, Greece; ^2^ Department of Respiratory Medicine, General Hospital of Pyrgos “Andreas Papandreou”, Pyrgos, Greece; ^3^ Allergy Centre, Wythenshawe Hospital, Manchester University NHS Foundation Trust, Manchester, United Kingdom; ^4^ Klarman Cell Observatory, Broad Institute of MIT and Harvard, Cambridge, MA, United States; ^5^ Division of Respiratory Medicine, Department of Internal Medicine, Patras University Hospital, Patras, Greece; ^6^ Division of Infectious Diseases, Department of Internal Medicine, Patras University Hospital, Patras, Greece; ^7^ Division of Endocrinology, Department of Internal Medicine, Patras University Hospital, Patras, Greece

**Keywords:** lymphocytes, T cells, Tregs, cytokines, transcription factors ROR-γt/Tbet/GATA-3/FoxP3/Ets-2, strenuous exercise

## Abstract

**Aim:**

Marathon is a running event in which athletes must cover a distance of 42.195 km. In addition to participating in marathons, marathoners have incorporated extensive running into their lifestyle. In the present study, we investigated the effect of long-term strenuous exercise in the form of marathon running on the immune system.

**Methods & Results:**

We collected peripheral blood samples from 37 male marathoners before/after a race and 37 age/sex/body mass index (BMI)-matched healthy sedentary controls. Hematological and biochemical tests revealed race-induced leukocytosis attributable to neutrophilia and significant increases in plasma lactate dehydrogenase (LDH), creatine phosphokinase (CPK), and cortisol concentrations. Phenotypic analysis of lymphocytes revealed race-induced significant decrease in the number of lymphocytes, memory helper T (Th) cells, naive, memory and activated cytotoxic T (Tc) cells, natural killer (NK), NKT, and B1 cells, and a significant increase in the number of activated Th and regulatory Th cells (Tregs). Compared with controls, marathoners maintained significantly lower levels of memory and activated Th cells and higher levels of activated Tc and B1 cells. Measurement of plasma cytokine levels revealed a pro-inflammatory cytokine polarization that increased after the race. Examination of gene expression of cytokines and Th-cell signature transcription factors in peripheral blood mononuclear cells revealed a significant decrease in tumor necrosis factor α (TNF-α) and interleukin (IL)-17, and a significant increase in IL-6, IL-10 and forkhead box P3 (FoxP3) after the race. Compared with controls, marathoners maintained significantly higher levels of TNF-α. Assessment of the suppressive capacity of Tregs in co-cultures of isolated effector Th cells and Tregs showed significantly increased suppressive capacity of marathoners’ Tregs after the race.

**Conclusions:**

Compared with controls, marathoners live with permanent changes in certain immune parameters. Marathoners exhibit a stable pro-inflammatory cytokine polarization that increases after the race and is counterbalanced by increased numbers of Tregs overexpressing FoxP3 and having increased suppressive capacity.

## Introduction

Regular moderate physical training has been shown to protect against morbidity and mortality associated with cardiovascular disease, type II diabetes mellitus, and cancer risk (1, 2). In addition, regular physical training has been shown to contribute to better health and less disability in the elderly by positively affecting immune function (3, 4). Regular moderate physical training has been reported to have anti-inflammatory effects due to the reduction of visceral fat mass and the induction of circulating anti-inflammatory cytokines (5, 6). These changes are clinically reflected in a lower incidence of infections (7, 8). In fact, moderate physical training has been reported to be associated with a significant reduction in the risk of respiratory infections compared to a sedentary lifestyle (9).

On the other hand, prolonged periods of strenuous physical training lead to a subclinical systemic inflammatory syndrome (SIRS) characterized by elevated levels of pro- and anti-inflammatory cytokines (10, 11). It has been suggested that strenuous exercise may cause suppression of immune function, making the host more susceptible to infection for 3 to 72 hours after exercise (“open window” theory) (7, 12, 13). In addition, acute physical training appears to increase oxidative stress and promote the production of reactive oxygen species (ROS), which is an important etiologic factor in diseases such as diabetes, cancer, and Parkinson’s disease (14, 15). Conditions associated with significant changes in stress system activity, such as acute or chronic stress and vigorous exercise, may increase autoimmune disease activity by modulating the systemic or local balance between pro- and anti-inflammatory cytokines (16). The above findings suggest that the type and form of exercise have different effects on the immune response.

The origins of marathon running date back to 490 BC. The name of the event comes from Philippides, who is said to have run from Marathon to Athens to bring news of the Greek victory at the Battle of Marathon. Centuries later, the popularity of the sport has not only diminished, but has actually increased dramatically in terms of the women to men ratio, age, and nationality of the participants (17).

Marathon runners are endurance athletes who are subjected to constant physical exertion because they not only participate in marathons but have integrated running into their lifestyle. During a marathon race, they may suffer from dehydration, hypothermia or hyperthermia, musculoskeletal, gastrointestinal and renal problems, and hyponatremia. Pulmonary complications may also occur, possibly associated with hyponatremia (18). Cardiac problems (occasionally severe) may occur, especially in athletes with congenital or acquired heart disease. In rare cases, marathon runners may experience exercise-induced anaphylaxis (18).

Marathon runners may develop upper respiratory tract infections (URTI) after a race, probably due to elevated serum cortisol levels, which have anti-inflammatory and immunosuppressive effects, and to desiccation of the mucosal surface of the respiratory tract as a result of the high ventilation rate during the race and inhalation of allergens or pollutants. Reactivation of viruses already present in the organism or infection with airborne viruses, usually rhinoviruses, in crowded venues could also contribute to the development of URTI after a race (13, 19–23).

As mentioned above, marathon runners have incorporated extensive running into their lifestyle in addition to participating in marathons. It is not clear whether endurance sports such as marathons have long-term effects on the immune system. In the present experimental study, we examined parameters of the immune system of habitual marathon runners to investigate whether and how long-term strenuous physical training in the form of marathon running affects the immune system and, in particular, T-cell-mediated immunity.

## Materials and methods

**Figure 1:**
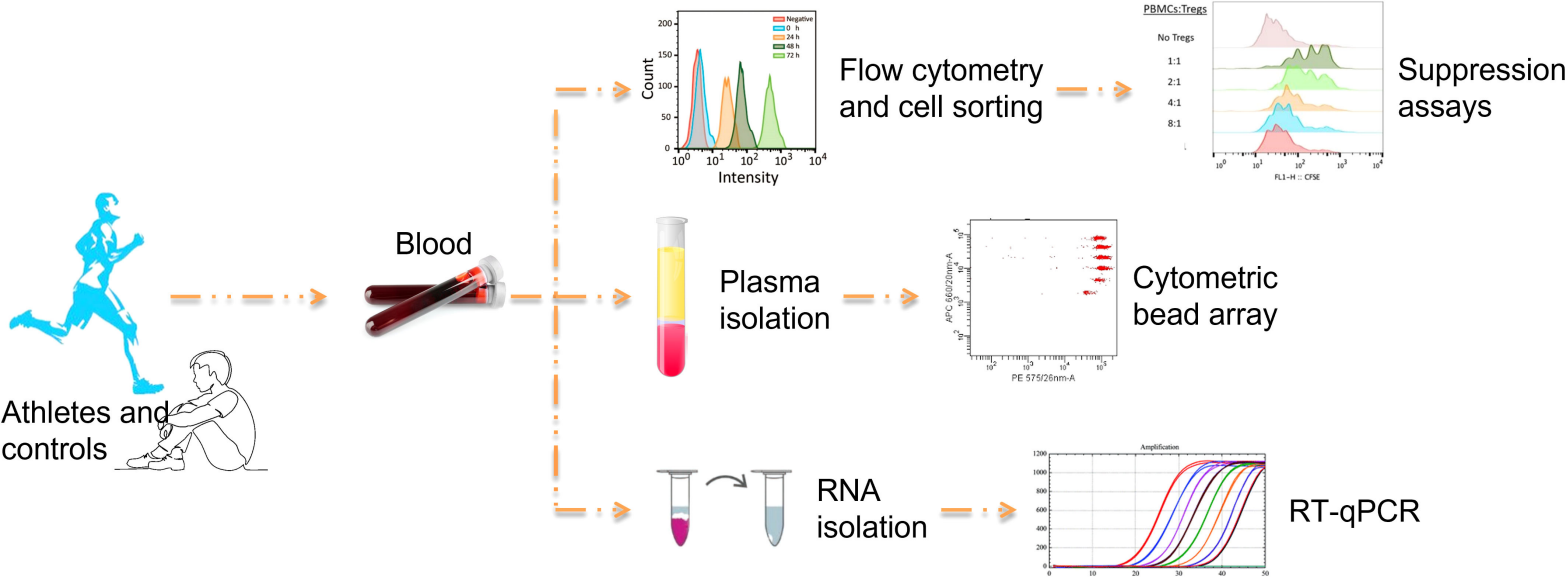
The experimental design of the study.

The experimental design of the study is illustrated in **Figure 1**.

### Study subjects

Thirty-seven male marathon runners volunteered for this study (mean age 50.2 ± 6.17 years, range 35-71 years, mean BMI 23.92 ± 1.51 kg/m^2^, range 20.14-24.72 kg/m^2^). The inclusion criteria were: (i) male marathon runners, (ii) who had completed an average of 11 classic marathons (42.195 km) and had been running long distances regularly for an average of 20 ± 10.4 years. Their routine training consisted of running (62.72 ± 9.58 km distance running/week) at varying paces, depending on the time of the official marathon. They were nonsmokers and had no history of febrile illness in the month before the race. None reported taking vitamins, minerals or other medications known to affect immune and circulatory function. None reported taking anti-inflammatory medications before, during, or after the race. None had an adverse medical event that required medical attention during or after the race. One day before the race, the marathon runners consumed the usual high-carbohydrate diet and abstained from alcohol and caffeine. The control group consisted of 37 age- and BMI-matched, apparently healthy men who did not engage in regular physical training (mean age 47.1 ± 5.31 years, range 35-68 years, mean BMI 24.31 ± 1.96 kg/m^2^, range 20.56-26.65 kg/m^2^). The characteristics of the marathon runners and controls are shown in **Table 1**.

**Table 1:** Characteristics of the study subjects.

	Marathon runners	Control group
n	37	37
age (yr)	50.2 ± 6.17, (35-71)	47.1 ± 5.31, (35-68)
BMI (kg/m^2^)	23.92 ± 1.51, (20.14-24.72)	24.31 ± 1.96, (20.56-26.65)
years of regular exercise	20.6 ± 10.4	–
RVW (km)	62.72 ± 9.58	–
MRPT (h)	4.41 ± 0.56	–
Classic marathons completed	11 ± 2.10	–

n, sample size; age in years, shown as mean ( ± SD), (range); BMI, body mass index (kg/m2 where kg is a person’s weight in kilograms and m2 is their height in meters squared), shown as mean ( ± SD), (range); years of regular exercise, shown as mean ( ± SD); RVW, running volume per week, shown as mean ( ± SD); MRPT, marathon personal record time, shown as mean ( ± SD). Symbol (–) means not applicable.

All study participants provided written informed consent before participating in the study. The study protocol was approved by the Scientific Review Board and the Ethics Committee of Patras University Hospital (PUH) (Re: 23/13.07.2016). PUH adheres to the Declaration of Helsinki on the Ethical Principles for Medical Research Involving Human Subjects.

### Blood samples

Blood samples (3-4 ml) were collected in the appropriate tubes for routine hematological and biochemical tests to determine the concentration of hemoglobin, hematocrit, white blood cells, C-reactive protein, glucose, lactate dehydrogenase, creatinine, uric acid, creatine phosphokinase, and cortisol.

Blood samples (3-4 ml) were collected in EDTA-coated tubes for flow cytometric analysis and determination of cytokine concentrations in plasma.

Blood samples (10 ml) were collected in heparin-coated tubes for isolation of peripheral blood mononuclear cells (PBMCs) for PCR analysis and cell sorting.

### Flow cytometry and cell sorting

Concentrations of lymphocyte populations in peripheral blood were determined in whole blood using the following mouse anti-human monoclonal antibodies: CD3-PC5 (Beckman Coulter-BC), CD4-FITC (BC), CD8-FITC (BC), CD45RA-PE (BC), CD45RO-PE (BC), CD20-PE (BC), CD5-FITC (BC), CD56-PE (BC), CD69-PE (Immunotech), CD25-PE (Becton Dickinson-BD), HLA-DR-PE (BD), CD25-PE (BD), FoxP3-PE-Cy5 (eBiosciences). All procedures were performed according to the manufacturer’s instructions. For intracellular staining of FoxP3, cells were labeled with CD4-FITC and CD25-PE antibodies and stained intracellularly for Foxp3 after fixation and permeabilization. Negative controls consisted of isotype-matched irrelevant antibodies replacing the specific antibodies at equivalent concentrations. Whole blood samples were treated with BD Pharm Lyse buffer 1x (BD) for 15 min after staining to lyse red blood cells. Flow cytometry was performed using the EPICS Coulter XL-MCL flow cytometer. At least 20,000 events were acquired for extracellular and 100,000 events for intracellular staining. Data analysis was performed using FlowJo V7.5 software (Tree Star Inc.).

**Figure 2:**
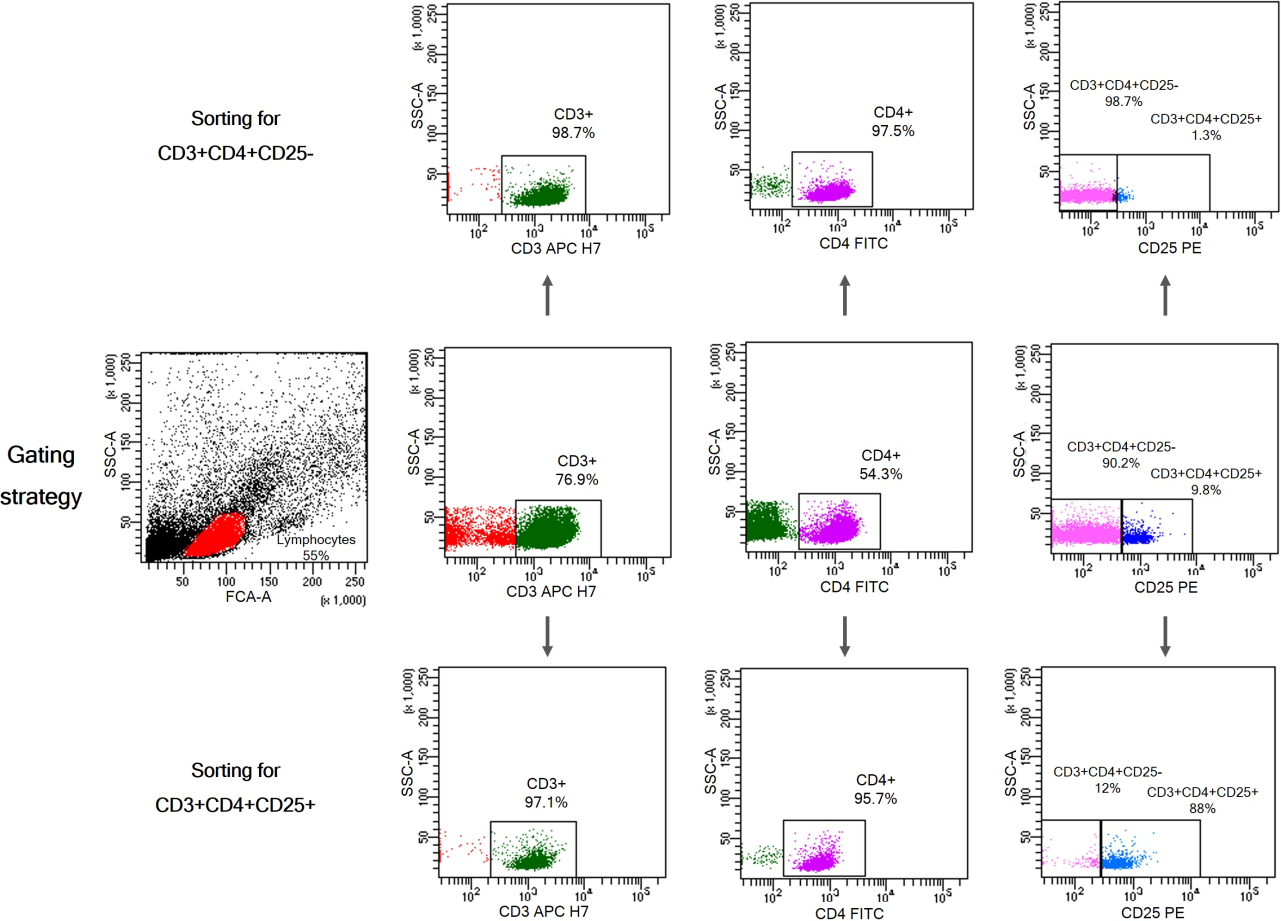
Gating strategy for isolation of CD3+CD4+CD25+ T cells and CD3+CD4+CD25- T cells from PBMCs. Characteristic dot plots showing purity after cell sorting using BD FACS Aria III Cell Sorter.

For cell sorting, PBMCs were isolated from whole blood by centrifugation over a Ficoll-Paque gradient (Biochrom), washed x4 in ice-cold RPMI1640 culture medium (Gibco, ThermoFisher Scientific) and counted in a hematology analyzer (Cell-Dyn 3700, Abbott). CD3+CD4+CD25+ and CD3+CD4+CD25- T cells were isolated from PBMCs using the BD FACS Aria III Cell Sorter. The sorting strategy is shown in **Figure 2**. The antibodies used for cell sorting and phenotyping were mouse anti-human monoclonal antibodies CD3-APC-H7 (BD), CD4-FITC (BC), and CD25-PE (BD). Data were analyzed using BD FACS DIVA v.9 software.

### Determination of plasma cytokine levels

Plasma IFN-γ, TNF-α, IL-2, IL-4, IL-6, IL-10, and IL-17A concentrations were measured by flow cytometry using a cytometric bead array (CBA) assay (human Th1/Th2/Th17 Cytokine Kit, BD). Analysis was performed using a BD FACSArray Bioanalyzer. Plasma concentrations of IL-1β, soluble IL-2 receptor 2R α chain (IL-2R) and TGF-β1 were measured by ELISA using the Human IL-1β Quantikine ELISA kit, the Human IL-2Rα Quantikine ELISA kit, and the Human/Mouse/Rat/Porcine/Canine TGF-β1 Quantikine ELISA kit (all from R&D Systems). All procedures were performed according to the manufacturer’s instructions. All measurements were performed in triplicate.

### Quantitative real time PCR

The expression levels of the cytokine genes IL-2, IFN-γ, IL-4, IL-6, TNF-α, TGF-β1, IL-10 and IL-17 and those of the transcription factors ROR-γt, Tbet, GATA-3, FoxP3 και Ets-2 were determined by qPCR. Total RNA was extracted from PBMCs using TRIzol^®^ reagent (Invitrogen), according to the manufacturer’s protocol. Total RNA was then reverse transcribed into cDNA using the M-MLV Reverse Transcriptase Kit (Promega). The reaction mixture was incubated at 37°C for 15 min and then at 85°C for 5 sec to inactivate the reverse transcriptase. The qPCR was performed on the Mx3000P QPCR system (Agilent Technologies) using the 2X SYBR^®^ Green qPCR Master Mix (ThermoFisher Scientific). The thermocycling conditions used were as follows: Denaturation at 95°C for 30 sec followed by 30 cycles at 95°C for 15 sec, 55°C for 30 sec, and 72°C for 2 min, and a final extension at 72°C for 5 min. The primer sequences used are listed in **Table 2**. Relative gene expression analysis was performed by the 2^–ΔΔCq^ method using Mx3000P QPCR software (Agilent Technologies). β2-Microglobulin served as an internal reference gene. All measurements were performed in triplicate.

**Table 2:** Primer sequences used for qPCR.

Gene	Forward primer (5’→3’)	Reverse primer (5’→3’)	Product length (bp)
IL-2	CTCACCAGGATGCTCACATTTA	CCTCCAGAGGTTTGAGTTCTTC	97
IFN-γ	TGGCTTTTCAGCTCTGCATC	CCGCTACATCTGAATGACCTG	117
IL-4	ACTTTGAACAGCCTCACAGAG	TTGGAGGCAGCAAAGATGTC	74
IL-6	GCCAGAGCTGTGCAGATGAG	AGGAACTCCTTAAAGCTGCG	188
TNF-α	TGGTGGTGCCATCAGAGGG	GGCTGATGGTGTGGGTGAGG	102
TGF-β1	AGGACCTCGGCTGGAAGTGG	AGTTGGCATGGTAGCCCTTG	51
IL-10	GCTGGAGGACTTTAAGGGTTACCT	CTTGATGTCTGGGTCTTGGTTCT	109
IL-17	CCCGGACTGTGATGGTCAAC	GCACTTTGCCTCCCAGATCA	158
ROR-γt	CTGCTGAGAAGGACAGGGAG	AGTTCTGCTGACGGGTGC	202
Tbet	TCCTGTTCCCAGCCGCTTCT	TGCTGACTGCTCGAAACTCA	122
GATA-3	GGGCTCTATCACAAAATGAACG	TTGTGGTGGTCTGACAGTTCGC	112
FoxP3	CGGACCATCTTCTGGATGAG	TTGTCGGATGATGCCACAG	172
Ets-2	CTCGTGTGTCTCAACCATCTT	CGCTCTGTGCCTCAGAATAG	112
β2m	TCGCGCTACTCTCTCTTTCT	TTTCCATTCTCTGCTGGATGAC	83

### Assays for Treg suppression function

The function of Tregs was validated by suppression assays using isolated CD3+CD4+CD25+ Tregs and CD3+CD4+CD25- effector Th cells (Teffs) from peripheral blood of marathon runners before and after a race and from control subjects. The suppressive activity of Tregs was assessed by measuring their ability to inhibit the activation and proliferation of activated Teffs. For this purpose, Teffs were incubated with CFSE (CellTrace™ CFSE Cell Proliferation Kit for flow cytometry, ThermoFisher Scientific) according to the manufacturer’s instructions and cultured in the presence of 10μg/ml phytohemagglutinin (PHA, from Sigma Aldrich) and Tregs (10,000 cells per experimental point) at the following Treg:Teff ratios: 0:1, 1:1, 1:2, 1:4, 1:8, for 72h. The proliferation of Teffs was measured by flow cytometry using the EPICS Coulter XL-MCL flow cytometer. Data analysis was performed using FlowJo V7.5 software (Tree Star Inc.).

### Statistical analysis

Data are expressed as mean (SD) and were analyzed with GraphPad Prism 6 (GraphPad Software, Inc.). Clinical parameters of the three groups (control vs. before race vs. after race) were compared using the nonparametric Kruskal-Wallis test for continuous variables because the data were not normally distributed. Comparisons between the three groups were made using the rank-based two-way ANOVA test followed by a Dunn’s multiple comparisons *post-hoc* test. Comparisons between data with two skewed distributions were made using the Mann-Whitney U test. Sample size was calculated using the equation from the power analysis of the diagnostic test (shown in **Supplementary Materials**). P<0.05 was considered to indicate a statistically significant difference.

## Results

### Hematological and biochemical parameters

**Table 3** shows the results of the hematological and biochemical tests performed on the blood samples of the marathon runners before and after the race. After the race, there was a significant increase in white blood cell (WBC) and neutrophil levels (for both p<0.05; H values: 6.483 and 8.794, respectively) and a significant decrease in lymphocyte, eosinophil, and monocyte levels (for all p<0.05; H values: 9.538, 12.05 and 6.537, respectively). In addition, LDH, CPK, and plasma cortisol levels were significantly elevated (for all p<0.05; H values: 11.93, 7.565 and 8.79, respectively). Comparisons between marathon runners before the race and controls showed that marathon runners maintained significantly higher levels of CPK (p<0.05; H value: 7.565).

**Table 3:** Laboratory parameters of marathon runners.

Laboratory data		Marathon runners (n=37)		Controls (n=37)	Normal range
		Before marathon	After marathon		
WBC	(K/μl)	6.0 (1.7)	13.9 (2.8)*	7.6 (3.4)	4-11
Neutrophils	(%)	53.1 (15.1)	77.23 (11.5)*	58.9 (9.5)	50-70
Lymphocytes	(%)	32.3 (12.0)	14.6 (9.8)*	31.2 (8.5)	20-40
Monocytes	(%)	9.6 (3.05)	6.7 (1.47)*	7.1 (1.4)	0-8
Eosinophils	(%)	3.7 (2.82)	0.64 (1.28)*	2.7 (2.2)	0-6
Basophils	(%)	0.34 (0.25)	0.52 (0.43)	0.34 (0.2)	0-1
Hemoglobin	(g/dl)	13.9 (0.5)	14.56 (9.4)	13.9 (1.7)	11.8-17.0
Hematocrit	(%)	41.6 (1.4)	43.14 (2.8)	41.7 (4.2)	36-52
LDH	(IU/l)	192.8 (49.2)	306.9 (99.1)*	171.4 (70.6)	120-230
CPK	(IU/l)	234.7 (175.2)**^#^**	417.5 (268.8)*	80.4 (13.6)	<190
Glucose	(mg/dl)	106.2 (10)	103 (20.1)	97.5 (19.2)	75-115
Creatinine	(mg/dl)	1.0 (0.14)	1.3 (0.18)	0.85 (0.22)	0.9-1.6
Uric Acid	(mg/dl)	4.8 (1.16)	5.8 (0.92)	3.6 (0.55)	3-7
CRP	mg/dl	<0.5	<0.5	<0.5	<0.5
Cortisol	(μg/dl)	14.6 (5.9)	25.8 (8.6)*	18.6 (9.9)	5-23

Data are shown as mean (SD); WBC, white blood cells; LDH, lactate dehydrogenase; CPK, creatine phosphokinase; CRP, C-reactive protein; asterisks denote statistically significant differences (*p<0.05) among hematological and biochemical parameters of marathoners before and after the race; number sign (#) denotes statistically significant difference (p<0.05) between CPK levels of controls and marathon runners before the race.

### Phenotypic analysis of peripheral blood lymphocytes

**Figure 3:**
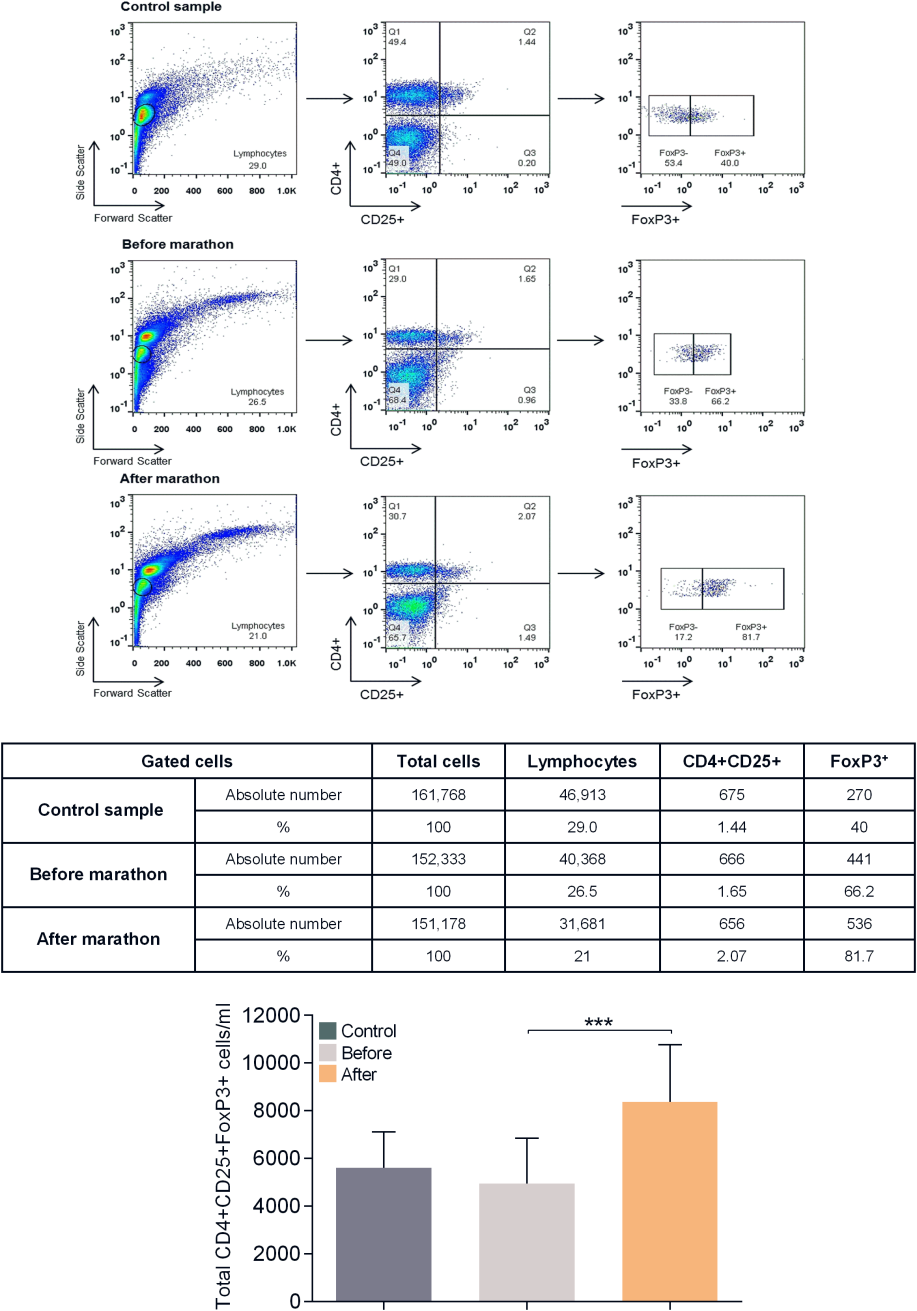
Flow cytometric analysis for determination of CD4+CD25^high^FoxP3+ Treg levels in human peripheral blood. Shown is a representative analysis for a control and a marathon runner before and after the race. Leukocytes were gated for lymphocytes using forward and side light scatter and analyzed for CD4 and CD25 expression. The double positive cells were additionally analyzed for FoxP3 expression. The numbers in the dot plots indicate the percentage of gated cells expressing the relevant marker. The table below shows the absolute number of cells in each population analyzed. Bottom graph: Results of the analysis of all study participants (control values are shown in dark gray, values of marathon runners before the race are shown in light gray, values of marathon runners after the race are shown in orange). Data are shown as mean (SD); ***p<0.001.

**Table 4** and **Figure 3** show the results of the phenotypic analysis performed on the blood samples of the marathon runners before and after the race and those of controls. Comparison of the values obtained from the marathon runners before and after the race showed that after the race there was a significant decrease in the concentration of lymphocytes, memory Th cells, total, naive, memory, and activated Tc cells, NK and NKT cells, and CD5+ B (B1) cells (all p<0.05; H values: 9.562, 14.86, 9.667, 11.19, 13.12, 9.768, 10.46, 7.764 and 13.65, respectively), as well as the ratio of Th to Tc cells (CD4/CD8) (p<0.05; H value: 12.86) (**Table 4**), whereas the concentration of Tregs increased significantly (p<0.001; H value: 22.18) (**Figure 3**).

**Table 4:** Cell surface immunophenotyping of peripheral blood lymphocytes of marathon runners vs. controls.

Cells	Marathon runners (n=37)		Controls (n=37)
	Before marathon	After marathon	
Lymphocytes (number of cells x10^7^/L)	185 (81)	162 (43)*	218 (53)
CD3+	76.8 (4.9)	56.8 (4.8)	72.3 (4.9)
CD3+CD4+	48.1 (4.5)	46.3 (4.7)	47.1 (4.9)
CD4+CD45RA+	22.2 (3.9)	25.9 (3.6)	19.0 (4.4)
CD4+CD45RO+	27.6 (1.2)	21.6 (1.2)*	30.2 (4.6)
CD4+CD69+	0.32 (0.03)	0.86 (0.08)*	0.2 (0.08)
CD4+HLA-DR+	0.76 (0.07)**^#^**	0.8 (0.08)	1.18 (0.3)
CD3+CD8+	29.8 (1.8)	14.8 (1.2)*	25.6 (4.6)
CD8+CD45RA+	20 (1.2)	9.2 (1.3)*	18 (3.3)
CD8+CD45RO+	12.4 (1.2)	4.3 (0.2)*	11.14 (3)
CD8+CD69+	1.1 (0.1)**^#^**	0.4 (0.03)*	0.31 (0.11)
CD8+HLA-DR+	0.4 (0.06)	0.25 (0.03)	0.58 (0.16)
CD3+CD56+	3.8 (0.6)	1.23 (0.2)*	3.6 (2)
CD3-CD56+	13.0 (0.7)	3.1 (0.3)*	9.6 (2.2)
CD20+	10.3 (0.6)	9.3 (0.5)	12.2 (2.2)
CD20+CD5+	1.2 (0.11)**^#^**	0.8 (0.07)*	0.61 (0.2)
CD4/CD8	3.32 (1.4)**^#^**	1.97 (0.77)*	1.93 (0.73)
CD4CD45RA/CD4CD45RO	1.04 (0.58)**^#^**	0.82 (0.59)	0.71 (0.38)
CD8CD45RA/CD8CD45RO	2.64 (1.86)	1.78 (0.57)	2.03 (0.98)

Data are shown as mean (SD); values represent % of lymphocytes; asterisks denote statistically significant differences (p<0.05) among lymphocyte populations (or ratios thereof) of marathoners before and after the race; number signs (#) denote statistically significant differences (p<0.05) between lymphocyte populations of controls and marathon runners before the race.

Comparisons between marathon runners before the race and controls showed that marathon runners had significantly lower levels of memory and long-term activated Th cells (both p<0.05; H values: 14.86 and 8.424, respectively), significantly higher levels of activated Tc cells and B1 cells (both p<0.05; H values: 9.768 and 13.65, respectively), and significantly higher ratios of Th/Tc and naïve/memory Th cells (both p<0.05; H values: 12.66 and 9.761, respectively) (**Table 4**).

### Plasma cytokine levels

**Figure 4:**
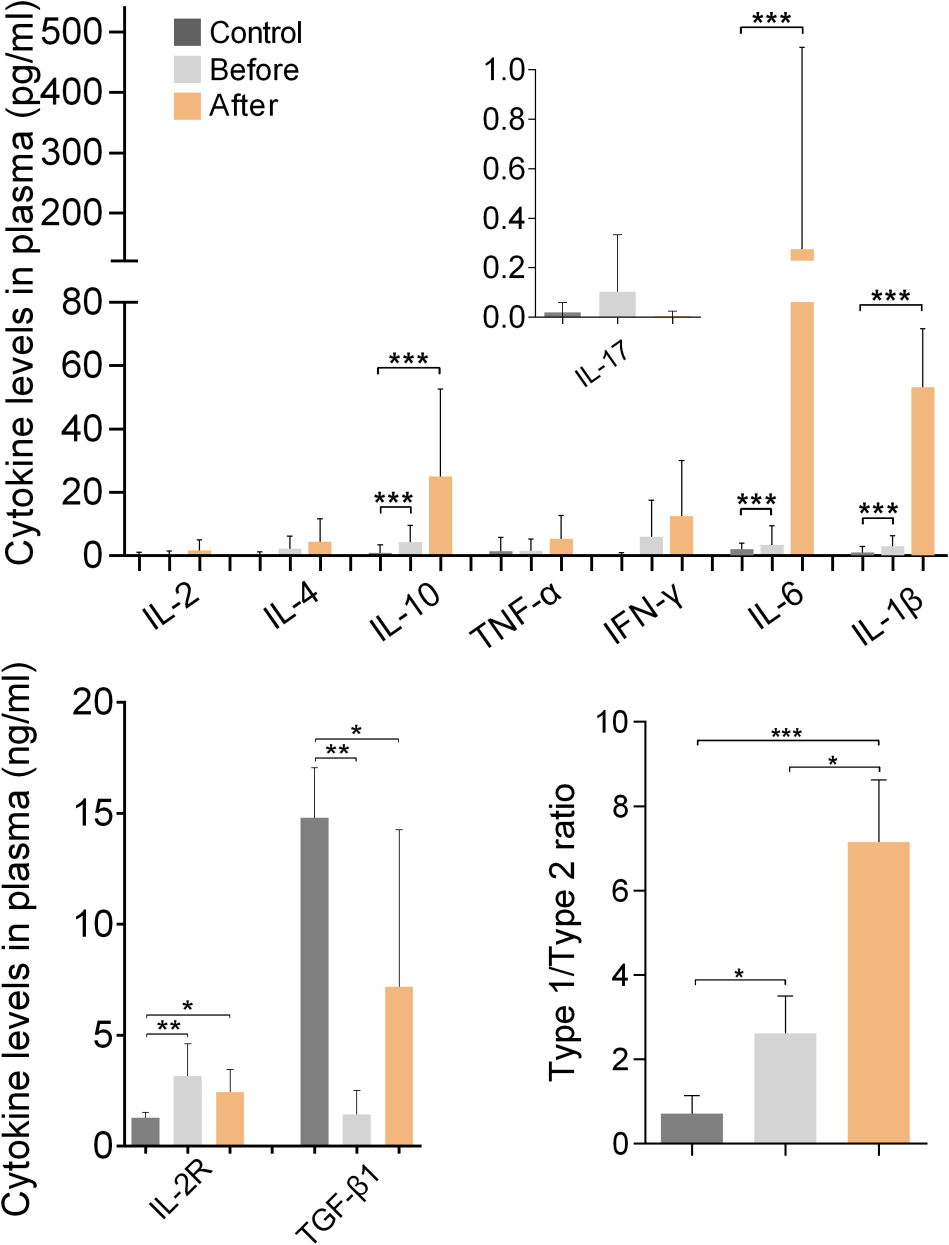
Plasma cytokine and sIL-2R levels of marathon runners before and after the race and of control subjects. Cytokine concentrations are shown as mean (SD). The concentration of IL-17 was measured in fg/ml and is shown in pg/ml. Type-1/type-2 cytokine ratio: [IL-1β+IL-2+IL-6+IL-17+IFN-γ+TNF-α]:[IL-4+IL-10+TGF-β1]. *p<0.05, **p<0.01, ***p<0.001.

**Figure 4** shows the results of measurements of cytokines and IL-2R in the plasma of marathon runners before and after the race and of controls. Comparison of the values obtained from marathon runners before and after the race revealed that after the race there was a significant increase in the concentrations of IL-1β, IL-6 and IL-10 (all p<0.001; H values: 22.07, 39.37 and 24.24, respectively), and a significantly increased type-1/type-2 (pro-inflammatory/anti-inflammatory) cytokine ratio (p<0.05 compared with the before group; p<0.01 compared with the control group; H value: 21.56).

Marathon runners had significantly higher levels of IL-2R and significantly lower levels of TGF-β1 than the control group both before and after the race (both p<0.01 compared with the before group; p<0.05 compared with the control group; H values: 13.99 and 12.96, respectively). In addition, marathon runners had a significantly increased type-1/type-2 cytokine ratio before the race compared with controls (p<0.05; H value: 26.51).

### Cytokine gene expression

**Figure 5:**
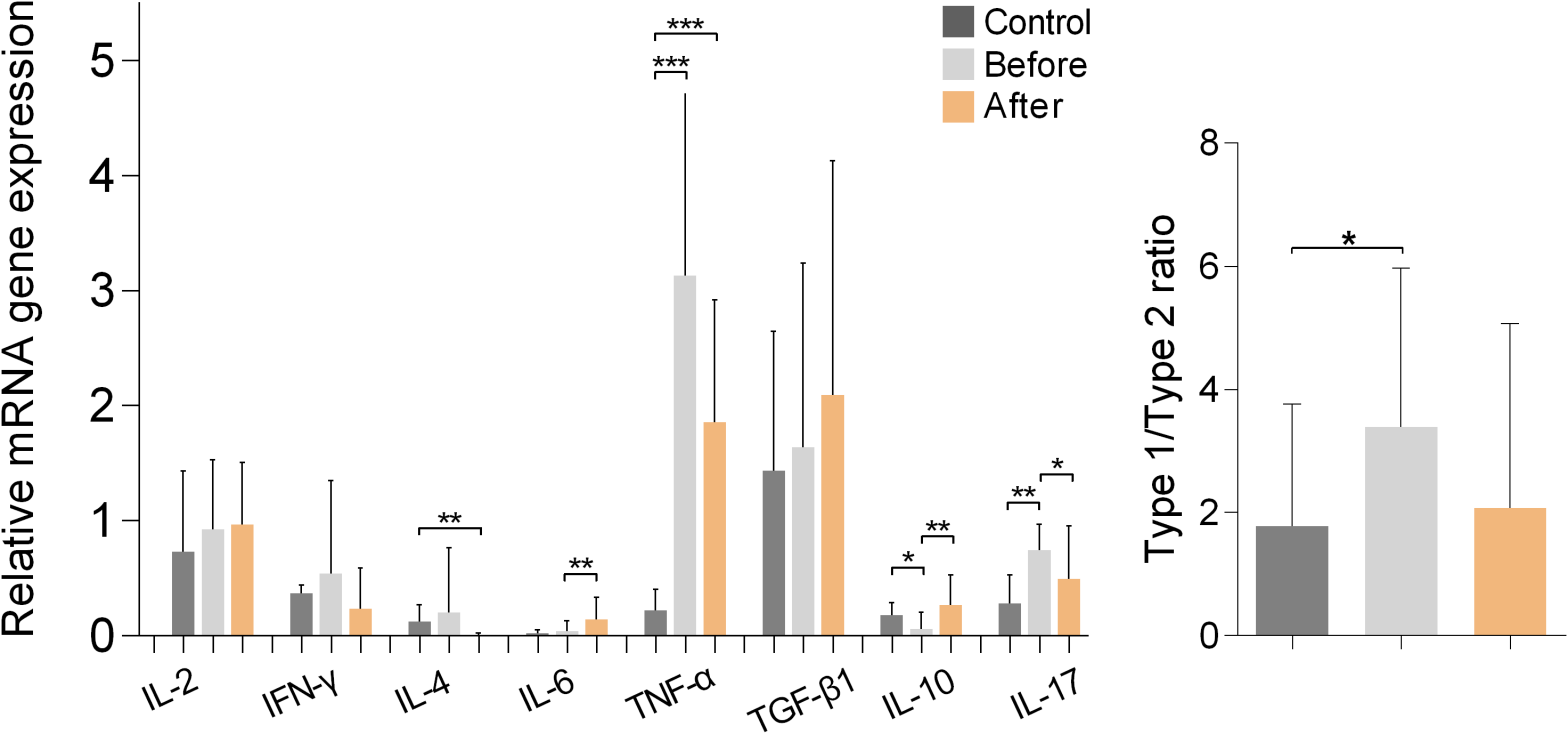
Relative mRNA gene expression of cytokines in PBMCs from marathon runners before and after the race and from control subjects. Data are shown as mean (SD). Type-1/type-2 ratio: [IL-2+IL-6+IL-17+IFN-γ+TNF-α]:[IL-4+IL-10+TGF-β1]. *p<0.05, **p<0.01, ***p<0.001.

**Figure 5** shows the results of measurements of cytokine gene expression in PBMCs of marathon runners before and after the race and of the controls. Comparison of the values obtained from the marathon runners before and after the race showed that after the race there was a significant increase in the expression of IL-6 and IL-10 (both p<0.01; H values: 9.334 and 14.14, respectively) and a significant decrease in the expression of IL-17 (p<0.05; H value: 17.72).

Comparisons between marathon runners and controls showed that marathon runners had significantly higher expression of TNF-α before and after the race (both p<0.001; H value: 20.27), whereas marathon runners significantly lower expression of IL-10 (p<0.05; H value: 14.14) and significantly higher expression of IL-17 before the race (p<0.01; H value: 17.72). In addition, marathon runners had significantly higher type-1/type-2 cytokine expression ratio before the race compared with controls (p<0.05; H value: 8.772).

### Expression of Th-tropic transcription factors

**Figure 6:**
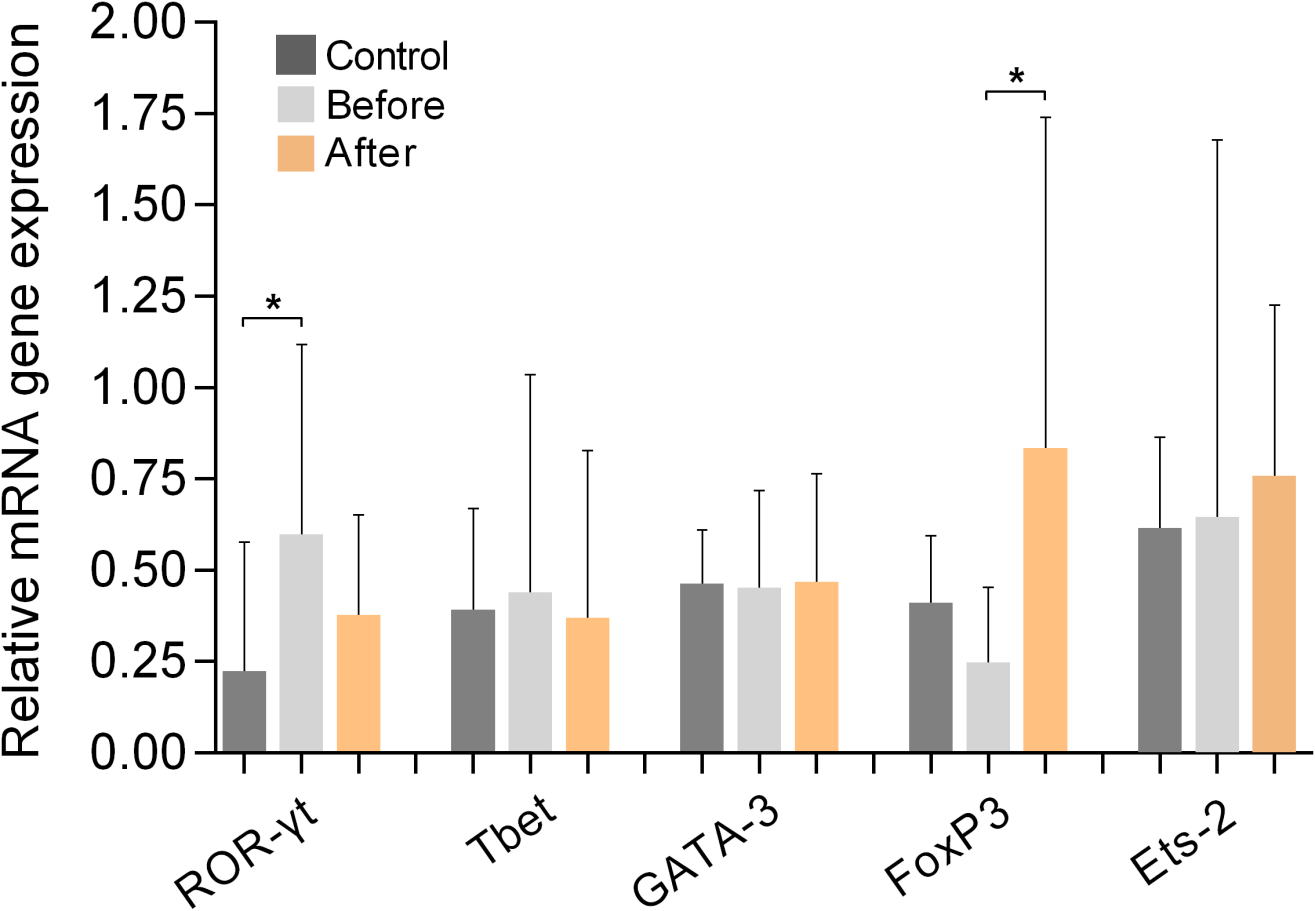
Relative mRNA gene expression of signature transcription factors of Th cell populations ROR-γt (Th17), Tbet (Th1), GATA-3 (Th2), FoxP3 (Tregs), and Ets-2 (pre-Th0) in PBMCs from marathon runners before and after the race and from control subjects. Data are shown as mean (SD). *p<0.05.

**Figure 6** shows the results of measurements of the expression of the Th-signature transcription factors Tbet (for Th1 cells), GATA-3 (for Th2 cells), ROR-γt (for Th17 cells), FoxP3 (for Tregs), and Ets-2 (naive, pre-Th0 cells) in PBMCs from marathon runners before and after the race and from controls. Comparison of the values obtained from marathon runners before and after the race revealed that the expression of FoxP3 was significantly increased after the race (p<0.05; H value: 9.518). In addition, ROR-γt was upregulated in marathon runners before the race compared with the control group (p<0.05; H value: 8.249). However, no significant differences in the mRNA expression levels of ROR-γt were found between the marathon runners in the “before” and “after” groups. The mRNA expression levels of Tbet, GATA-3, and Ets-2 remained unchanged in the three groups.

### Function of Tregs

**Figure 7:**
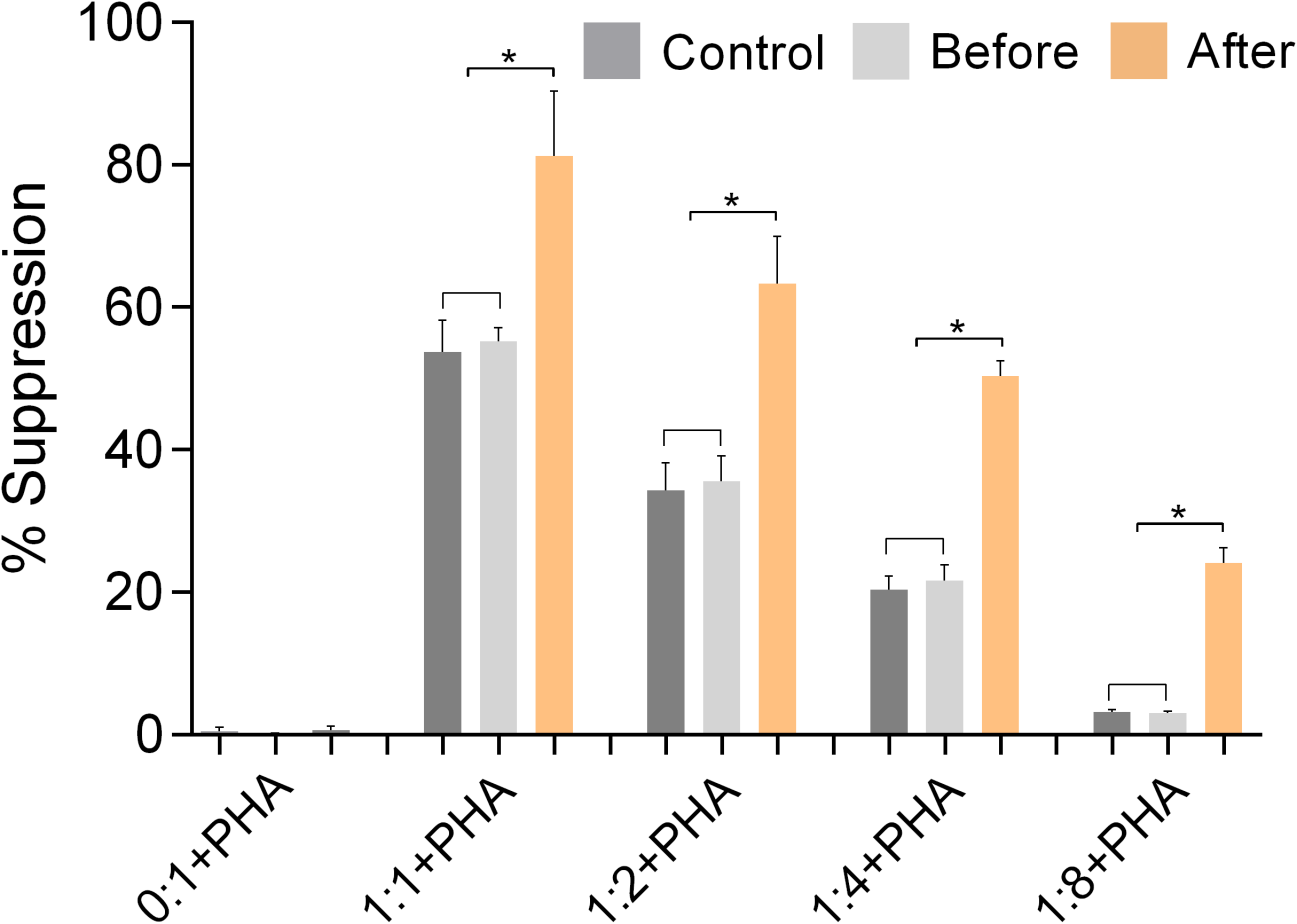
The function of Tregs was validated by suppression assays using isolated CD3+CD4+CD25+ Tregs and CD3+CD4+CD25- effector Th cells (Teffs) from PBMCs of marathon runners before and after a race and from control subjects. The suppressive activity of Tregs was assessed by measuring their ability to inhibit the activation and proliferation of activated Teffs (% suppression). Teffs were incubated with CFSE and cultured in the presence of PHA and Tregs at the following ratios of Tregs to Teffs: 0:1, 1:1, 1:2, 1:4, 1:8. Data are expressed as mean (SD). *p<0.05.

**Figure 7** shows the results of experiments conducted to evaluate the ability of isolated Tregs to inhibit the activation and proliferation of isolated Teffs. The results show that Tregs isolated from marathon runners after the race had significantly increased suppressive capacity (p<0.05), whereas Tregs isolated from marathon runners before the race had a suppressive function similar to control Tregs.

## Discussion

The results of this study show that there are both transient and permanent changes in various immune parameters in marathon runners.

Among the laboratory parameters studied, we found leukocytosis due to neutrophilia and a significant decrease in the concentrations of eosinophils, monocytes, and lymphocytes as well as increased cortisol levels in marathon runners after the race, which is consistent with previous studies, except for monocytes (21, 22, 24, 25). CPK was the only parameter that was consistently elevated in marathon runners compared to control subjects, indicating progressive muscle damage in the period leading up to a marathon, as marathon runners have integrated running into their lifestyle (26).

Detailed phenotypic analysis of peripheral blood lymphocytes revealed significant decreases in memory Th cells, Tc cells (naive, memory, and activated), NK cells, NKT cells, B1 cells, and Th:Tc ratio, and significant increases in activated Th cells and Tregs after running. Values for controls (**Table 4**) were consistent with those reported in previous published studies in healthy individuals (27, 28).

Comparisons between marathon runners before the race and controls showed that marathon runners had significantly lower levels of activated Th cells, significantly higher levels of activated Tc and B1 cells, and significantly higher ratios of Th:Tc cells and naive:memory Th cells.

Both the transient and the permanent changes in the leukocyte and lymphocyte populations of marathon runners do not support the assumption that strenuous training leads to a deterioration of host defenses. On the contrary, the increased levels of activated Tc in marathon runners compared to sedentary controls suggest that this form of exercise enhances the Tc branch of adaptive immunity that protects against viral infections or cancer (29, 30). In addition, significantly increased levels of B1 cells (and the antibodies they produce) contribute to better host defenses, particularly against bacteria (31, 32).

Measurement of plasma cytokines revealed that running a marathon significantly affected the balance between type-1and type-2 cytokines. After the marathon, both the pro-inflammatory cytokines IL-1β and IL-6, and the anti-inflammatory cytokine IL-10 were elevated. Plasma levels of IL-17 were very low compared with the other cytokines measured, and there were no statistically significant differences in IL-17 plasma levels among the three groups. These results suggest that plasma levels of Il-17 were not affected in marathon runners and are consistent with previous studies showing that serum or plasma levels of IL-17 remained unchanged in athletes participating in endurance sports such as marathons (33–35).

In contrast to previous studies (12, 36–39), increased cortisol levels did not suppress type-1 cytokine production, and increased IL-6 levels did not stimulate type-2 cytokine production or inhibit TNF-α production. Instead, a strong type-1 cytokine polarization was observed. This type-1 polarization was reduced in marathon runners before the race but remained significantly higher than in sedentary control subjects, mainly due to significantly lower TGF-β1 levels and significantly higher sIL-2R levels. The increased sIL-2R levels in marathon runners before the race, in conjunction with our data showing similar levels of IL-2 and Tregs compared with controls, suggest that sIL-2R is secreted to capture and remove IL-2 from the circulation. This is supported by our results showing that the levels of IL-2 and sIL-2R do not increase significantly after the race, i.e., they do not correlate with the increased number of Tregs, suggesting that the IL-2/sIL-2R pathway does not have a tolerogenic function in marathon runners (40).

Measurement of cytokine expression in PBMCs isolated from marathon runners and control subjects revealed significant type-1 cytokine polarization in marathon runners before the race, indicating an established constitutive pro-inflammatory cytokine profile. This type-1 cytokine polarization increases significantly after the race and is counterbalanced by an increase in Treg numbers and secretion of IL-10.

Measurement of Th cell signature transcription factor expression (41–45) revealed a significant increase in FoxP3 after the race that correlated with increased expression and secretion of IL-10, as well as significantly higher levels of ROR-γt before the race compared with controls. The expression levels of ROR-γt correlated with the expression and secretion levels of IL-17. This anti-diametric expression of FoxP3 and ROR-γt may be due to an inhibitory effect that FoxP3 exerts on ROR-γt expression (46).

Finally, our results from the functional assays performed with isolated effector Th cells and Tregs showed that the significantly increased expression of FoxP3 after the race correlated with a significantly increased suppressive capacity of Tregs (44). The increase in FoxP3 after racing is likely due to increased cortisol levels, which have been shown to increase FoxP3 expression (47).

## Conclusions and future perspectives

Considering that the parameters measured in marathon runners before a race represent the steady state of their immune system, their immune system seems to function in a different steady state than that of healthy, sedentary control subjects and is characterized by pro-inflammatory cytokine polarization, which is also observed in certain type-1 autoimmune diseases, with many important changes in their cellular immunity including overactive Tregs. This altered immune system is observed in habitual marathon runners regardless of age, and it appears to function well. To note, our data suggest that marathon running induces a subclinical SIRS characterized by hypercytokinemia and increased WBC levels. Specifically, our results showed stable pro-inflammatory (type-1) cytokine polarization in marathon runners, which increased markedly after the race and was counterbalanced by a transient increase in Treg numbers and activity, and secretion of IL-10. Thus, our results do not suggest suppression of the immune system but rather successful regulation that occurs after a race when the pro-inflammatory cytokine polarization is maximized.

Because the current study examined a homogeneous group of marathon runners in terms of sex, age, BMI, weekly running distance, and years of marathon running, it was not possible to correlate specific aspects of marathon running, such as elite professional vs. amateur marathon runners, runners with different BMI ranges, age (young vs. older runners), individual marathon performance rates, etc., with specific parameters of the immune system that were investigated in the present study. As a possible limitation of the study, we acknowledge that due to the rather limited sample size, we were only able to reliably detect strong effects (as indicated by the power analysis and *post-hoc* power analysis, see **Supplementary Materials** for more details).

This work sets the stage for future studies with a larger sample size, that will examine the above parameters, and, also, investigate what happens to marathon runners when they stop training; specifically, whether their immune system reverts to that of healthy sedentary control subjects or whether they develop chronic inflammatory conditions.

## Data availability statement

The raw data supporting the conclusions of this article will be made available by the authors, without undue reservation.

## Ethics statement

The study protocol was approved by the Scientific Review Board and the Ethics Committee of Patras University Hospital (PUH) (Re: 23/13.07.2016). PUH adheres to the Declaration of Helsinki on the Ethical Principles for Medical Research Involving Human Subjects. The patients/participants provided their written informed consent to participate in this study.

## Author contributions

NC and AM designed the study. NC, KK, LL, NG, KM and AM were responsible for recruitment of study participants, approval of the study protocol, and collection of biological samples. IP, PS, A-LL, MR, SA and IA performed the experiments. CL performed the power analysis. IP, IT, CL and AM analyzed the data. IP, IT and AM, prepared the figures, and wrote the manuscript. All authors were responsible for revising the manuscript for important intellectual content. All authors contributed to the article and approved the submitted version.
