# Supplementary material for: Shifting gears: Study of immune system parameters of male habitual marathon runners
Source: Front Immunol. 2023 Jan 13;13:1009065. doi: 10.3389/fimmu.2022.1009065 (PMC9880332; doi:10.3389/fimmu.2022.1009065)
Supplement: Supplementary file 2 [file DataSheet_2.zip › FI-1009065-R4-SUPPLEMENTARY MATERIALS-Kruskal_Wallis_and_posthoc_power_analysis/Kruskal_Wallis_and_posthoc_power_analysis.pdf]

In [37]:
```
# Load the required libraries/packages
suppressMessages(library(tidyverse))
library(rcompanion)
suppressMessages(library(rstatix))
suppressMessages(library(FSA))
suppressMessages(library(pwr)) # Required for power analysis
```

In [38]:
```
data_il4 <- suppressMessages(read_csv('IL4.csv'))
```

In [39]:
```
data_long_il4 <- data_il4 %>%
    pivot_longer(c(control, before, after))
```

In [40]:
```
colnames(data_long_il4) <- c('group','value')
```

In [41]:
```
# Specify functions for calculation of mean and standard deviation
mean_drop_na <- partial(mean, na.rm = TRUE)
sd_drop_na <- partial(sd, na.rm = TRUE)
```

In [42]:
```
# Calculate the mean and standard deviation per group (control, before marathon, after marathon)
data_long_il4 %>%
    group_by(group) %>%
    summarize(group_mean = mean_drop_na(value), group_sd = sd_drop_na(value))
```

A tibble: 3 × 3

group	group_mean	group_sd
<chr>	<dbl>	<dbl>
after	4.4231818	7.1160258
before	2.1809091	3.9864555
control	0.3486667	0.9213412

In [43]:
```
# Perform the Kruskal-Wallis test
res.kruskal.il4 <- data_long_il4 %>%
    kruskal_test(value ~ group)
res.kruskal.il4
```

A rstatix_test: 1 × 6

	.y.	n	statistic	df	p	method
	<chr>	<int>	<dbl>	<int>	<dbl>	<chr>
1	value	66	3.776504	2	0.151	Kruskal-Wallis

In [44]:
```
# Perform the Kruskal-Wallis test
kruskal.test(value ~ group, data = data_long_il4)
```

```
	Kruskal-Wallis rank sum test

data:  value by group
Kruskal-Wallis chi-squared = 3.7765, df = 2, p-value = 0.1513
```

Based on the Kruskal-Wallis test, no significant difference between the groups was detected, as p-value > 0.05.

The eta squared, based on the H-statistic, can be used to measure the effect size of the Kruskal-Wallis test. It is calculated based on the formula: eta2[H] = (H - k + 1)/(n - k); where H is the value obtained in the Kruskal-Wallis test; k is the number of groups; n is the total number of observations (Tomczak and Tomczak, 2014)

In [45]:
```
# Calculate the effect size
data_long_il4 %>%
    kruskal_effsize(value ~ group)
```

A rstatix_test: 1 × 5

	.y.	n	effsize	method	magnitude
	<chr>	<int>	<dbl>	<chr>	<ord>
1	value	66	0.02819848	eta2[H]	small

Given the small effect and that the power analysis we previously performed showed us that the sample size we have does not offer us enough power to detect statistical difference for such small effects, we perform post-hoc power analysis to see what power we achieve. Given that 'pwr.anova.test' from the pwr package works with a balanced design (equal sample size for all groups), we get the average number of observations per group as sample size: n = 66/3 = 22. Also, since ANOVA is the parametric equivalent and as a rule of thumb 15% increase in sample size is required to achieve the same power between the parametric and non-parametric test, we use n = 0.85 * 22 = 18.7, so approximately 19 observations per group.

In [59]:
```
pwr.anova.test(k = 3,
               f = 0.028,
               n = 19,
               sig.level = 0.05)
```

```
     Balanced one-way analysis of variance power calculation

              k = 3
              n = 19
              f = 0.028
      sig.level = 0.05
          power = 0.05318437

NOTE: n is number in each group
```

This post-hoc power analysis shows that the study is clearly underpowered to detect such small effects. This is in accordance with the previously performed power analysis.

We repeat the procedure for IL-10, where we found significant difference

In [15]:
```
data_il10 <- suppressMessages(read_csv('IL10.csv'))
```

In [16]:
```
data_long_il10 <- data_il10 %>%
    pivot_longer(c(control, before, after))
```

In [17]:
```
colnames(data_long_il10) <- c('group','value')
```

In [18]:
```
# Calculate the mean and standard deviation per group (control, before marathon, after marathon)
data_long_il10 %>%
    group_by(group) %>%
    summarize(group_mean = mean_drop_na(value), group_sd = sd_drop_na(value))
```

A tibble: 3 × 3

group	group_mean	group_sd
<chr>	<dbl>	<dbl>
after	25.0363636	27.640210
before	4.2904545	5.326084
control	0.9413333	2.423686

In [19]:
```
# Perform the Kruskal-Wallis test
kruskal.test(value ~ group, data = data_long_il10)
```

```
	Kruskal-Wallis rank sum test

data:  value by group
Kruskal-Wallis chi-squared = 24.237, df = 2, p-value = 5.457e-06
```

In [20]:
```
# Calculate the effect size
data_long_il10 %>%
    kruskal_effsize(value ~ group)
```

A rstatix_test: 1 × 5

	.y.	n	effsize	method	magnitude
	<chr>	<int>	<dbl>	<chr>	<ord>
1	value	66	0.3529727	eta2[H]	large

As expected, given that the effect size is large, post-hoc power analysis reveals that the power is now much larger compared to that achieved with the same sample size when the effect size was small

In [54]:
```
pwr.anova.test(k = 3,
               f = 0.353,
               n = 19,
               sig.level = 0.05)
```

```
     Balanced one-way analysis of variance power calculation

              k = 3
              n = 19
              f = 0.353
      sig.level = 0.05
          power = 0.6361871

NOTE: n is number in each group
```

Given that there are significant differences, we perform the Dunn's test as a post-hoc test, to find which groups ('control', 'before', or 'after' that correspond to the control group, marathoners before marathon and after marathon respectively) are different from each other. As there are multiple comparisons, we will use the Benjamini-Hochberg FDR method (5% cut-off)

In [21]:
```
dunnTest(value ~ as.factor(group), data = data_long_il10, method = "bh")
```

```
Warning message:
“Some rows deleted from 'x' and 'g' because missing data.”
Dunn (1964) Kruskal-Wallis multiple comparison

  p-values adjusted with the Benjamini-Hochberg method.

        Comparison        Z      P.unadj        P.adj
1   after - before 3.784533 1.539973e-04 0.0002309960
2  after - control 4.515453 6.318167e-06 0.0000189545
3 before - control 1.107668 2.680051e-01 0.2680050517
```

The Kruskal-Wallis test showed us that there is a significant difference in the IL-10 levels of the three groups (p-value = 5.46e-06). We observe that there is a significant difference in the levels of IL-10 for marathoners before and after the marathon (Dunn's Test, P.adj = 0.00023), as well as the levels of IL-6 of marathoners after the marathon and the control group (Dunn's Test, P.adj = 0.00002). However, there is no significant difference at the levels of IL-6 of marathoners before the marathon and the control group (Dunn's Test, P.adj = 0.26801).

We repeat the procedure for IL-6, where we found significant difference

In [28]:
```
data_il6 <- suppressMessages(read_csv('IL6.csv'))
```

In [29]:
```
data_long_il6 <- data_il6%>%
    pivot_longer(c(control, before, after))
```

In [30]:
```
colnames(data_long_il6) <- c('group','value')
```

In [31]:
```
# Calculate the mean and standard deviation per group (control, before marathon, after marathon)
data_long_il6 %>%
    group_by(group) %>%
    summarize(group_mean = mean_drop_na(value), group_sd = sd_drop_na(value))
```

A tibble: 3 × 3

group	group_mean	group_sd
<chr>	<dbl>	<dbl>
after	140.167727	334.569020
before	3.418182	5.997275
control	2.160769	1.839531

In [32]:
```
# Perform the Kruskal-Wallis test
kruskal.test(value ~ group, data = data_long_il6)
```

```
	Kruskal-Wallis rank sum test

data:  value by group
Kruskal-Wallis chi-squared = 39.367, df = 2, p-value = 2.828e-09
```

In [34]:
```
# Calculate the effect size
data_long_il6 %>%
    kruskal_effsize(value ~ group)
```

A rstatix_test: 1 × 5

	.y.	n	effsize	method	magnitude
	<chr>	<int>	<dbl>	<chr>	<ord>
1	value	66	0.5931313	eta2[H]	large

In this case, given that the effect size is even larger than in the case of IL-10, we expect that the post-hoc calculated power will be even larger. This is indeed the case

In [56]:
```
pwr.anova.test(k = 3,
               f = 0.593,
               n = 19,
               sig.level = 0.05)
```

```
     Balanced one-way analysis of variance power calculation

              k = 3
              n = 19
              f = 0.593
      sig.level = 0.05
          power = 0.9802175

NOTE: n is number in each group
```

Given that there are significant differences, we perform the Dunn's test as a post-hoc test, to find which groups ('control', 'before', or 'after' that correspond to the control group, marathoners before marathon and after marathon respectively) are different from each other. As there are multiple comparisons, we will use the Benjamini-Hochberg FDR method (5% cut-off)

In [35]:
```
dunnTest(value ~ as.factor(group), data = data_long_il6, method = "bh")
```

```
Warning message:
“Some rows deleted from 'x' and 'g' because missing data.”
Dunn (1964) Kruskal-Wallis multiple comparison

  p-values adjusted with the Benjamini-Hochberg method.

        Comparison         Z      P.unadj        P.adj
1   after - before  5.892735 3.798543e-09 1.139563e-08
2  after - control  4.483847 7.330917e-06 1.099638e-05
3 before - control -0.595052 5.518087e-01 5.518087e-01
```

The Kruskal-Wallis test showed us that there is a significant difference in the IL-6 levels of the three groups (p-value = 2.83e-09) We observe that there is a significant difference in the levels of IL-6 for marathoners before and after the marathon (Dunn's Test, P.adj = 1.14e-08), as well as the levels of IL-6 of marathoners after the marathon and the control group (Dunn's Test, P.adj = 1.1e-05). However, there is no significant difference at the levels of IL-6 of marathoners before the marathon and the control group (Dunn's Test, P.adj = 5.5e-01).

As far as the post-hoc power analysis is concerned, we conclude that in agreement with the power analysis we previously performed, the sample size we have is not enough to detect small effect sizes (and thus the study is underpowered to detect rather small differences), whereas the study has adequate power to detect large effects.

In [68]:
```
sessionInfo()
```

```
R version 4.2.2 (2022-10-31)
Platform: x86_64-apple-darwin13.4.0 (64-bit)
Running under: macOS Big Sur ... 10.16

Matrix products: default
BLAS/LAPACK: /Users/lazarch2/opt/anaconda3/envs/r_env_4/lib/libopenblasp-r0.3.21.dylib

locale:
[1] en_US.UTF-8/en_US.UTF-8/en_US.UTF-8/C/en_US.UTF-8/en_US.UTF-8

attached base packages:
[1] stats     graphics  grDevices utils     datasets  methods   base

other attached packages:
 [1] FSA_0.9.3         rcompanion_2.4.18 forcats_0.5.2     stringr_1.5.0
 [5] dplyr_1.0.10      purrr_1.0.0       readr_2.1.3       tidyr_1.2.1
 [9] tibble_3.1.8      ggplot2_3.4.0     tidyverse_1.3.2

loaded via a namespace (and not attached):
 [1] matrixStats_0.63.0  fs_1.5.2            bit64_4.0.5
 [4] lubridate_1.9.0     httr_1.4.4          repr_1.1.4
 [7] tools_4.2.2         backports_1.4.1     utf8_1.2.2
[10] R6_2.5.1            nortest_1.0-4       DBI_1.1.3
[13] colorspace_2.0-3    withr_2.5.0         tidyselect_1.2.0
[16] Exact_3.2           bit_4.0.5           compiler_4.2.2
[19] cli_3.5.0           rvest_1.0.3         expm_0.999-6
[22] xml2_1.3.3          sandwich_3.0-2      scales_1.2.1
[25] lmtest_0.9-40       mvtnorm_1.1-3       proxy_0.4-27
[28] multcompView_0.1-8  pbdZMQ_0.3-8        digest_0.6.31
[31] base64enc_0.1-3     pkgconfig_2.0.3     htmltools_0.5.4
[34] dunn.test_1.3.5     dbplyr_2.2.1        fastmap_1.1.0
[37] rlang_1.0.6         readxl_1.4.1        rstudioapi_0.14
[40] generics_0.1.3      zoo_1.8-11          jsonlite_1.8.4
[43] vroom_1.6.0         googlesheets4_1.0.1 magrittr_2.0.3
[46] modeltools_0.2-23   Matrix_1.5-3        Rcpp_1.0.9
[49] DescTools_0.99.47   IRkernel_1.3.1      munsell_0.5.0
[52] fansi_1.0.3         lifecycle_1.0.3     stringi_1.7.8
[55] multcomp_1.4-20     MASS_7.3-58.1       rootSolve_1.8.2.3
[58] plyr_1.8.8          grid_4.2.2          parallel_4.2.2
[61] crayon_1.5.2        lmom_2.9            lattice_0.20-45
[64] IRdisplay_1.1       haven_2.5.1         splines_4.2.2
[67] hms_1.1.2           pillar_1.8.1        uuid_1.1-0
[70] boot_1.3-28.1       gld_2.6.6           codetools_0.2-18
[73] stats4_4.2.2        reprex_2.0.2        glue_1.6.2
[76] evaluate_0.19       data.table_1.14.6   modelr_0.1.10
[79] vctrs_0.5.1         tzdb_0.3.0          cellranger_1.1.0
[82] gtable_0.3.1        assertthat_0.2.1    coin_1.4-2
[85] libcoin_1.0-9       broom_1.0.2         e1071_1.7-12
[88] class_7.3-20        survival_3.4-0      googledrive_2.0.0
[91] gargle_1.2.1        timechange_0.1.1    TH.data_1.1-1
[94] ellipsis_0.3.2
```
