# Supplementary material for: Shifting gears: Study of immune system parameters of male habitual marathon runners
Source: Front Immunol. 2023 Jan 13;13:1009065. doi: 10.3389/fimmu.2022.1009065 (PMC9880332; doi:10.3389/fimmu.2022.1009065)
Supplement: Supplementary file 1 [file analysis.pdf › analysis.pdf]

Statistical analysis

Table of contents

Statistical analysis **1**
Power analysis and sample size 1
Power analysis 2
Cohen effect sizes for t-test 2
Cohen effect sizes for ANOVA 3
Power of t-test and sample size 4
Small effect size 4
Medium effect size 6
Large effect size 8
Power of ANOVA and sample size 10
Small effect size 10
Medium effect size 12
Large effect size 14

Statistical analysis

In the Methods section of the manuscript, it was accidentally stated that the statistical tests used for comparison of two means and three means were Student's t-test and ANOVA, respectively. We thank the reviewer for helping us spot this. In reality, the tests used were their non-parametric counterparts Mann-Whitney and Kruskal-Wallis. Given that the non-parametric tests make no assumptions on normality, there is no need to check for normality. In addition, there is no need to check if the variance is the same across samples.

Power analysis and sample size

For calculating the required sample size, we performed power analysis based on the corresponding parametric test and then increased the required sample size by 15% (Guidance on

sample size calculation for non-parametric tests). For the power analysis, we used the "pwr" R package (CRAN page for "pwr"). We calculated the sample size for different Cohen effect sizes and power = 0.8. The steps of the analysis are shown below:

We install or load the required packages

```
required_packages <- c("pwr")
need_to_install <- setdiff(required_packages, rownames(installed.packages()))
```

```
install.packages(need_to_install)
```

We load the required packages

```
suppressMessages(library(pwr)) # Needed for power analysis
```

Power analysis

First, we calculate the small, medium, and large effect sizes for the t-test:

Cohen effect sizes for t-test

```
cohen_small_t <- cohen.ES(test = 't' , size = 'small')
cohen_small_t
```

```

     Conventional effect size from Cohen (1982)

           test = t
           size = small
    effect.size = 0.2
```

```
cohen_medium_t <- cohen.ES(test = 't' , size = 'medium')
cohen_medium_t
```

```

     Conventional effect size from Cohen (1982)

           test = t
           size = medium
    effect.size = 0.5
```

```r
cohen_large_t <- cohen.ES(test = 't' , size = 'large')
cohen_large_t
```

```
     Conventional effect size from Cohen (1982) 

           test = t
           size = large
    effect.size = 0.8
```

Cohen effect sizes for ANOVA

Then, we calculate small, medium, and large effect sizes for ANOVA:

```r
cohen_small_anova <- cohen.ES(test = 'anov' , size = 'small')
cohen_small_anova
```

```
     Conventional effect size from Cohen (1982) 

           test = anov
           size = small
    effect.size = 0.1
```

```r
cohen_medium_anova <- cohen.ES(test = 'anov' , size = 'medium')
cohen_medium_anova
```

```
     Conventional effect size from Cohen (1982) 

           test = anov
           size = medium
    effect.size = 0.25
```

```r
cohen_large_anova <- cohen.ES(test = 'anov' , size = 'large')
cohen_large_anova
```

```
     Conventional effect size from Cohen (1982)

           test = anov
           size = large
    effect.size = 0.4
```

Power of t-test and sample size

Now, we calculate the number of individuals for each group (n), in order to achieve statistical power of 0.8, for the different effect sizes and significance level 0.05

Small effect size

```r
# Power of two-sided, unpaired t-test for small Cohen effect
t_power_cs <- pwr.t.test(
    d = 0.2,
    sig.level = 0.05,
    type = c("two.sample"),
    alternative = c("two.sided"),
    power = 0.8
)
```

```r
t_power_cs
```

```
     Two-sample t test power calculation

              n = 393.4057
              d = 0.2
      sig.level = 0.05
          power = 0.8
    alternative = two.sided

NOTE: n is number in *each* group
```

```r
plot(t_power_cs)
```

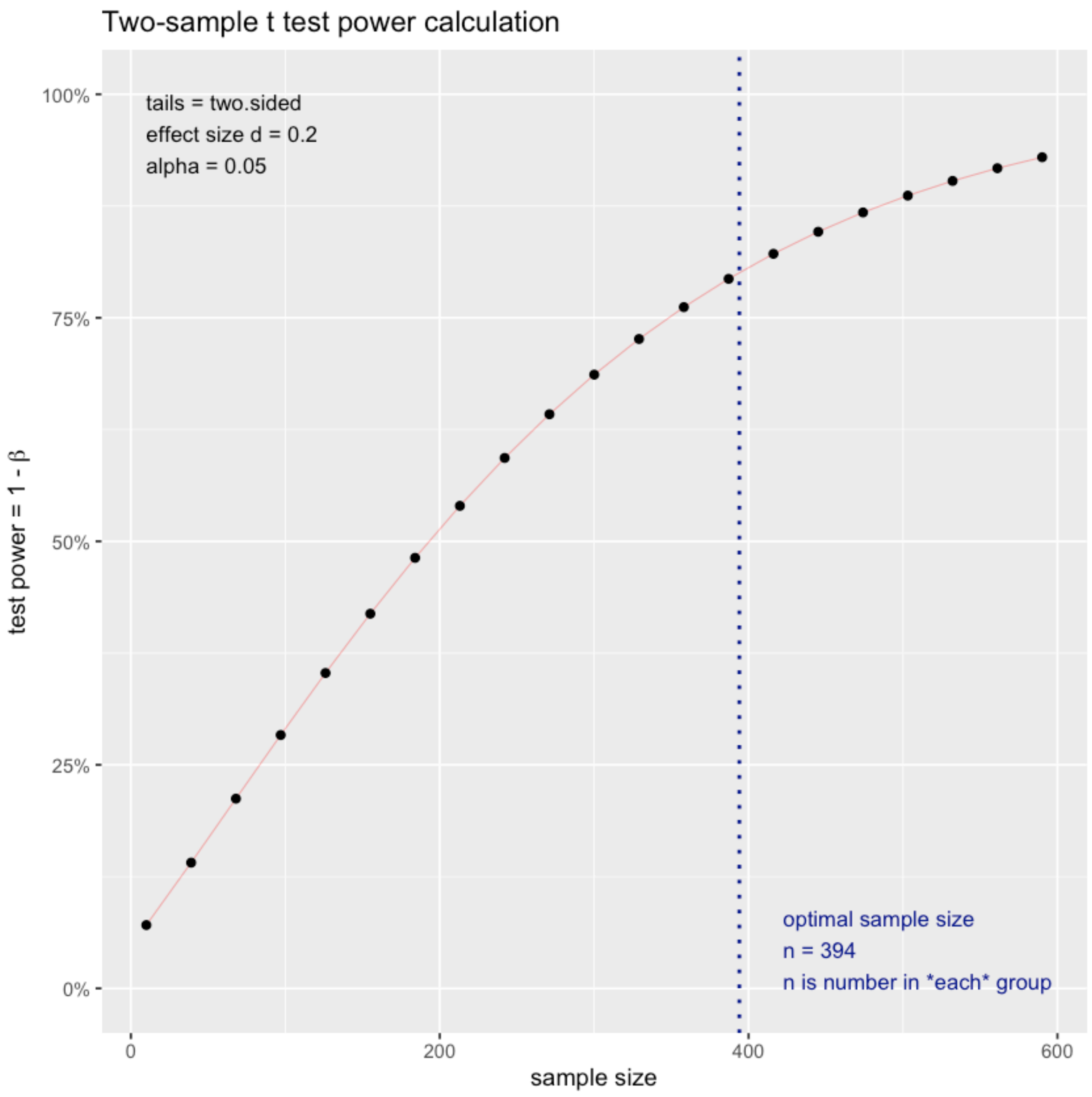

To achieve the same with the non-parametric equivalent (Mann-Whitney) that we used, we would need a sample size n=452 (per group), as shown below. We do not have that sample size

```
n = round(1.15*393)
n
```

452

Medium effect size

```r
# Power of two-sided, unpaired t-test for medium Cohen effect
t_power_cm <- pwr.t.test(
    d = 0.5,
    sig.level = 0.05,
    type = c("two.sample"),
    alternative = c("two.sided"),
    power = 0.8
)
```

```r
t_power_cm
```

```

     Two-sample t test power calculation

              n = 63.76561
              d = 0.5
      sig.level = 0.05
          power = 0.8
    alternative = two.sided

NOTE: n is number in *each* group
```

```r
plot(t_power_cm)
```

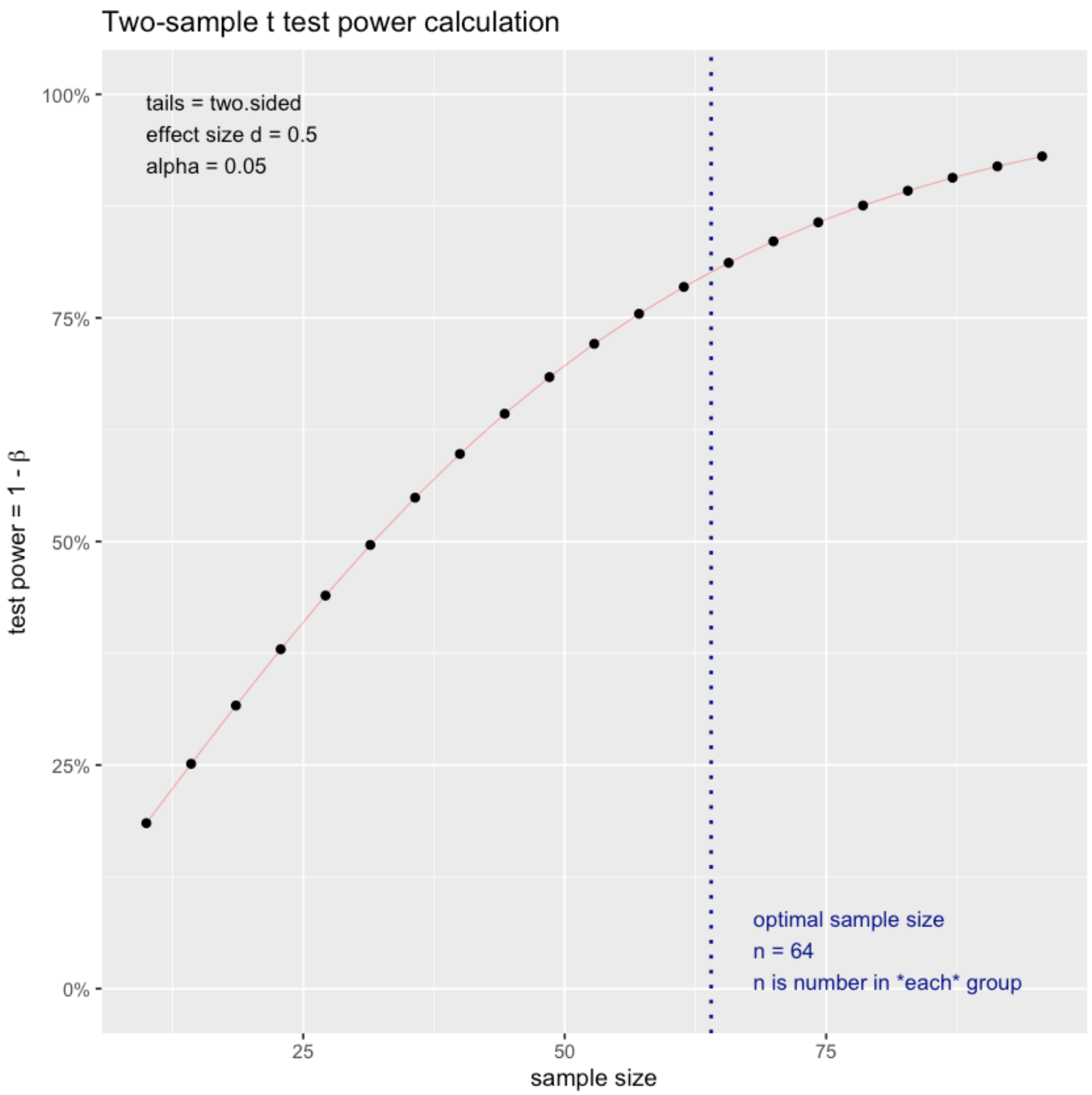

To achieve the same with the non-parametric equivalent (Mann-Whitney) that we used, we would need a sample size n=74 (per group), as shown below. We do not have that sample size

```
n = round(1.15*64)
n
```

74

Large effect size

```r
# Power of two-sided, unpaired t-test for large Cohen effect
t_power_cl <- pwr.t.test(
    d = 0.8,
    sig.level = 0.05,
    type = c("two.sample"),
    alternative = c("two.sided"),
    power = 0.8
)
```

```r
t_power_cl
```

```
     Two-sample t test power calculation

              n = 25.52458
              d = 0.8
      sig.level = 0.05
          power = 0.8
    alternative = two.sided

NOTE: n is number in *each* group
```

```r
plot(t_power_cl)
```

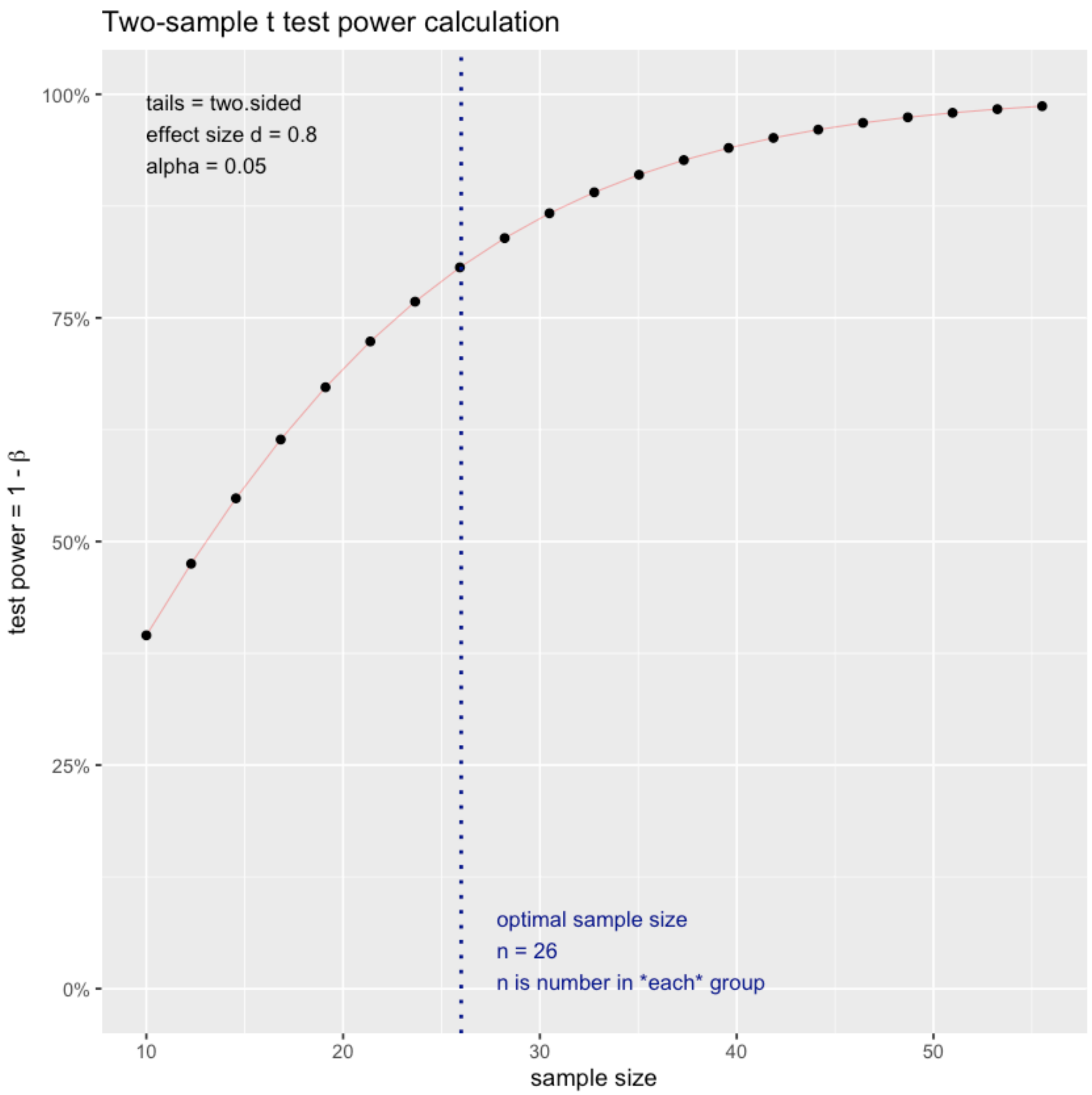

To achieve the same with the non-parametric equivalent (Mann-Whitney) that we used, we would need a sample size n=26 (per group), as shown below.

```
n = round(1.15*26)
n
```

30

With the sample size of the study, we should be able to detect large effects

Power of ANOVA and sample size

We repeat the procedure for ANOVA, as we examine the difference in means of three samples (k = 3). We calculate the number of individuals for each group (n), in order to achieve statistical power of 0.8 (power = 0.8), for the different effect sizes (small, medium, large), and significance level 0.05 (sig.level = 0.05)

Small effect size

```
# Power of ANOVA for small Cohen effect and 3 groups
anova_power_cs <- pwr.anova.test(
    k = 3,
    f = 0.1,
    sig.level = 0.05,
    power = 0.8
)
```

```
anova_power_cs
```

```

     Balanced one-way analysis of variance power calculation

              k = 3
              n = 322.157
              f = 0.1
      sig.level = 0.05
          power = 0.8

NOTE: n is number in each group
```

```
plot(anova_power_cs)
```

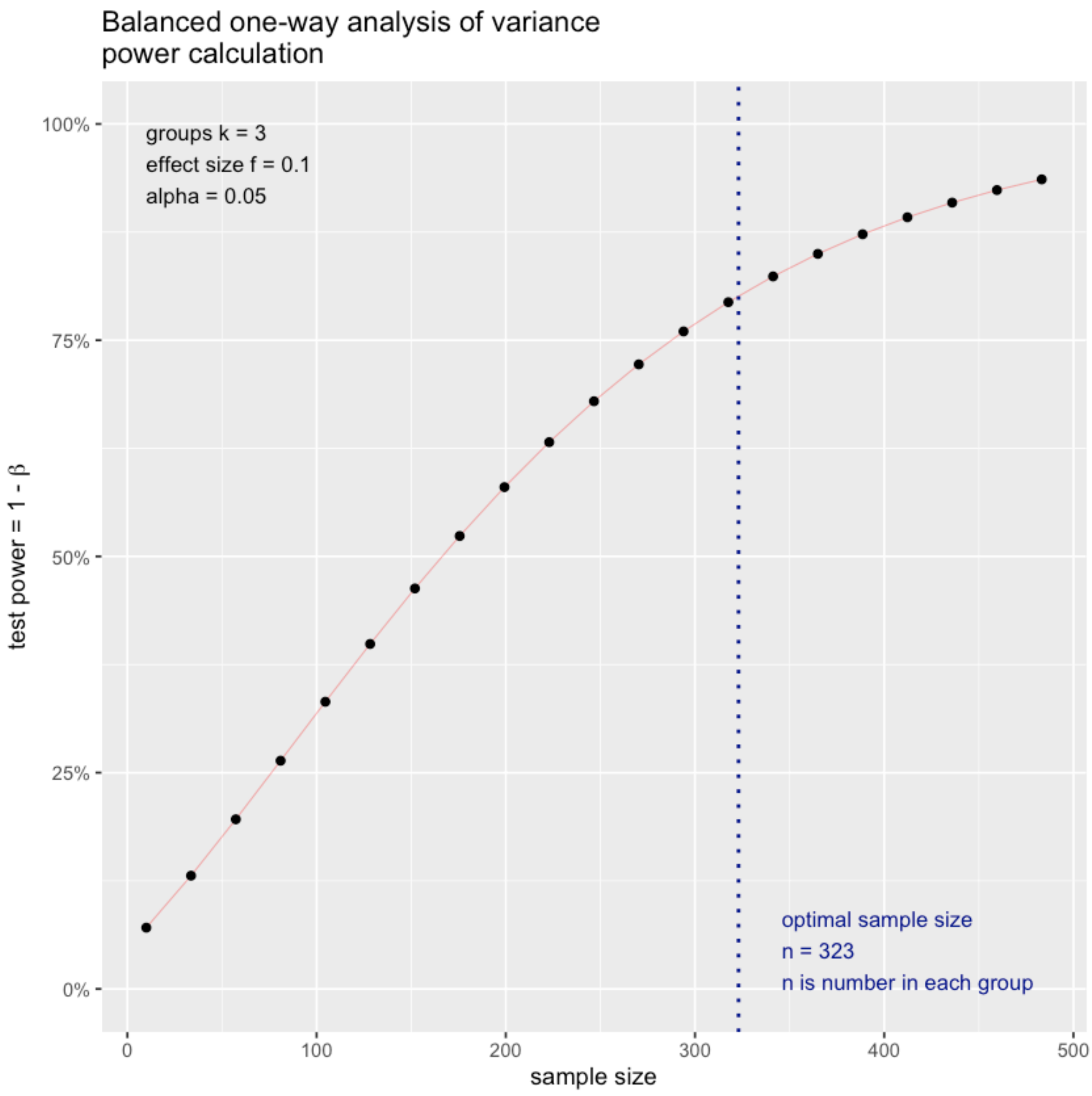

To achieve the same with the non-parametric equivalent (Kruskal-Wallis) that we used, we would need a sample size n=370 (per group), as shown below. We do not have that sample size.

```
n = round(1.15*322)
n
```

370

Medium effect size

```r
# Power of ANOVA for medium Cohen effect and 3 groups
anova_power_cm <- pwr.anova.test(
    k = 3,
    f = 0.25,
    sig.level = 0.05,
    power = 0.8
)
```

```r
anova_power_cm
```

```

     Balanced one-way analysis of variance power calculation

              k = 3
              n = 52.3966
              f = 0.25
      sig.level = 0.05
          power = 0.8

NOTE: n is number in each group
```

```r
plot(anova_power_cm)
```

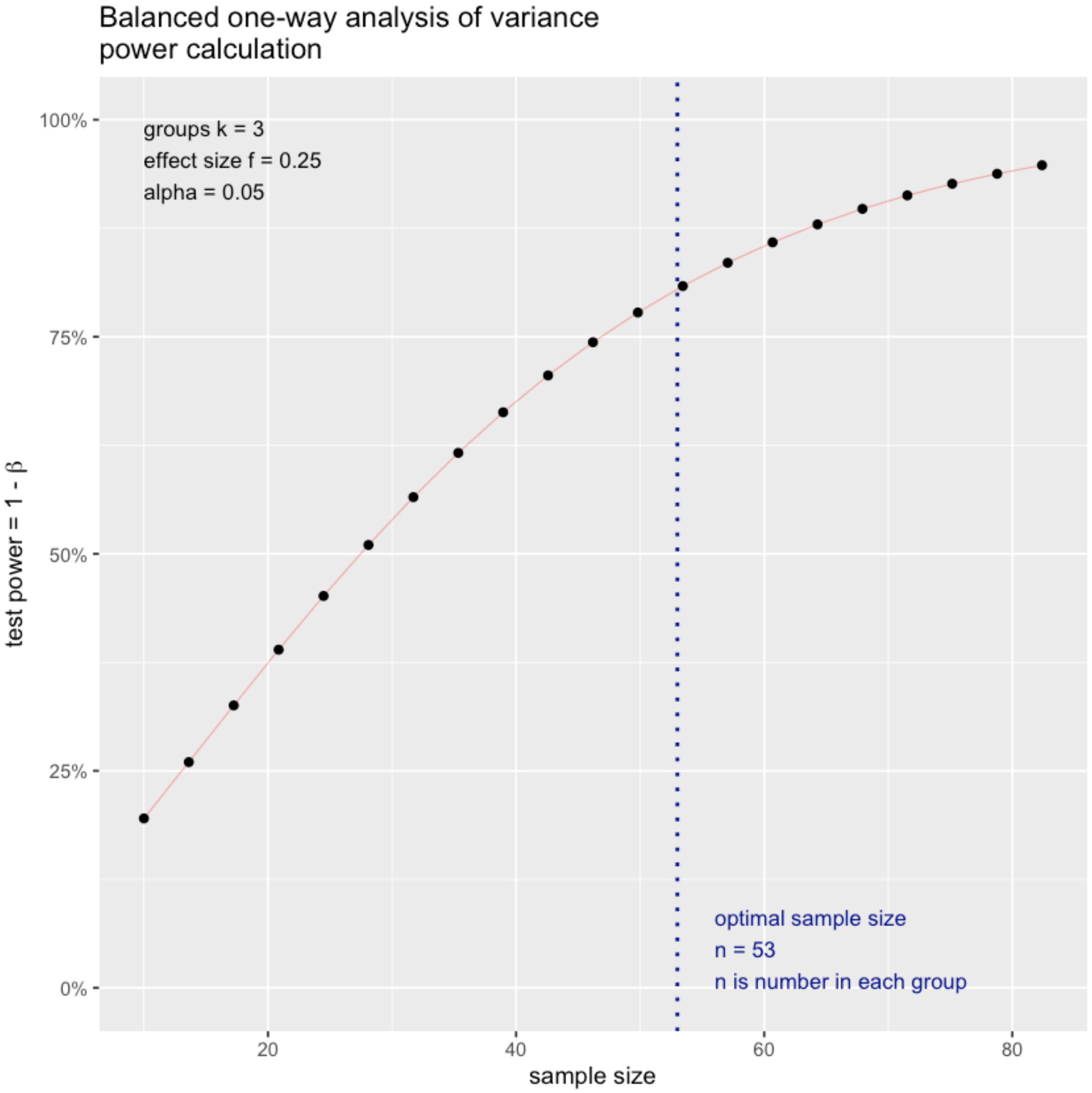

To achieve the same with the non-parametric equivalent (Kruskal-Wallis) that we used, we would need a sample size n=60 (per group), as shown below. We do not have that sample size.

```
n = round(1.15*52)
n
```

60

Large effect size

```r
# Power of ANOVA for large Cohen effect and 3 groups
anova_power_cl <- pwr.anova.test(
    k = 3,
    f = 0.4,
    sig.level = 0.05,
    power = 0.8
)
```

```r
anova_power_cl
```

```
     Balanced one-way analysis of variance power calculation 

              k = 3
              n = 21.10364
              f = 0.4
      sig.level = 0.05
          power = 0.8

NOTE: n is number in each group
```

```r
plot(anova_power_cl)
```

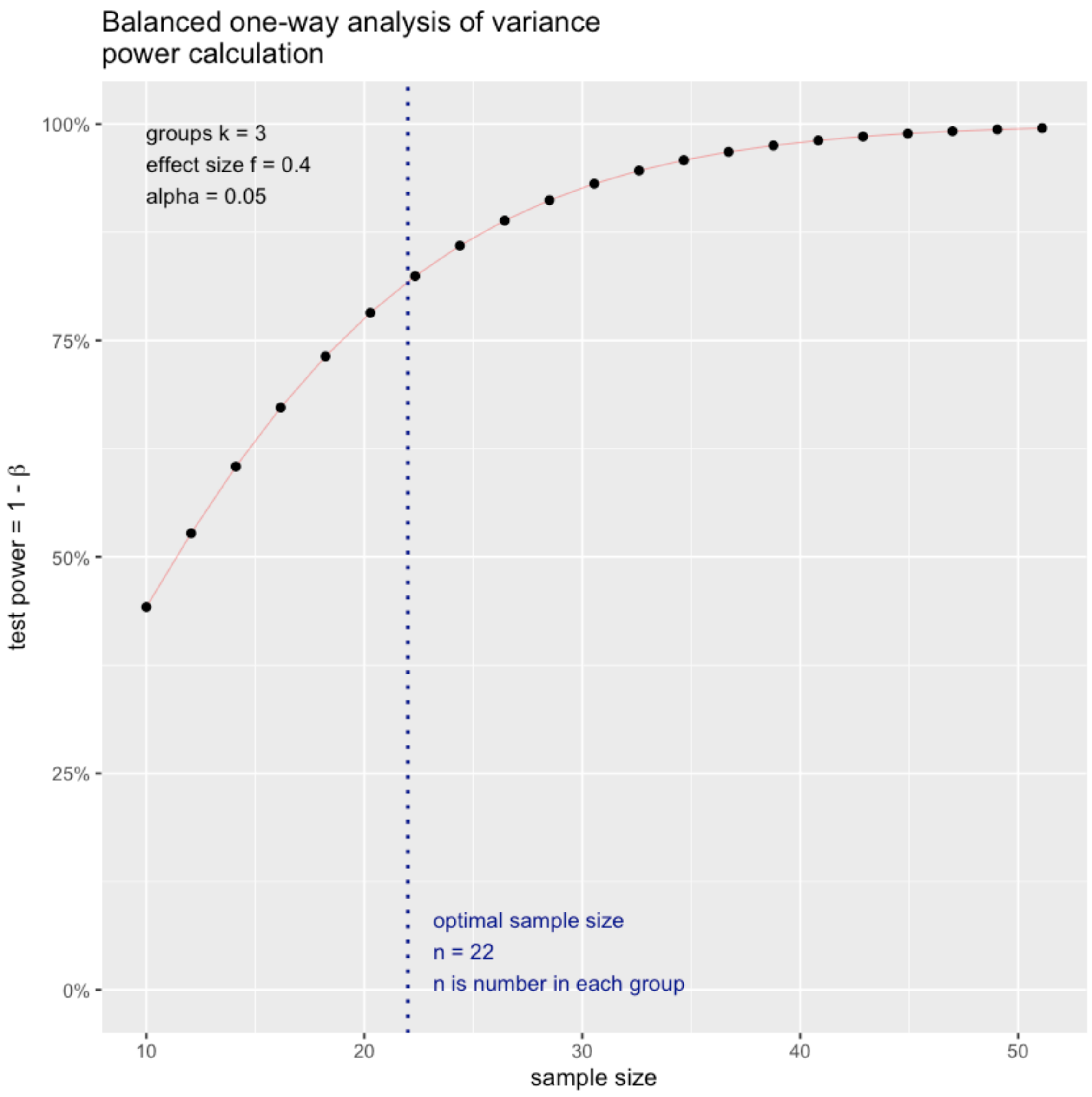

To achieve the same with the non-parametric equivalent (Kruskal-Wallis) that we used, we would need a sample size n=24 (per group), as shown below

```
n = round(1.15*21)
n
```

24

With the sample size of the study, we should be able to detect large effects

Here are all the details of the R session:

```r
sessionInfo()
```

```
R version 4.2.1 (2022-06-23)
Platform: x86_64-apple-darwin17.0 (64-bit)
Running under: macOS Big Sur ... 10.16

Matrix products: default
BLAS:   /Library/Frameworks/R.framework/Versions/4.2/Resources/lib/libRblas.0.dylib
LAPACK: /Library/Frameworks/R.framework/Versions/4.2/Resources/lib/libRlapack.dylib

locale:
[1] C/UTF-8/C/C/C/C

attached base packages:
[1] stats     graphics  grDevices utils     datasets  methods   base

other attached packages:
[1] pwr_1.3-0

loaded via a namespace (and not attached):
 [1] pillar_1.8.1      compiler_4.2.1    ggpubr_0.4.0      tools_4.2.1
 [5] base64enc_0.1-3   digest_0.6.30     uuid_1.1-0        evaluate_0.18
 [9] jsonlite_1.8.3    lifecycle_1.0.3   tibble_3.1.8      gtable_0.3.1
[13] pkgconfig_2.0.3   rlang_1.0.6       IRdisplay_1.1     DBI_1.1.3
[17] cli_3.4.1         IRkernel_1.3.1    fastmap_1.1.0     repr_1.1.4
[21] dplyr_1.0.10      generics_0.1.3    vctrs_0.5.0       grid_4.2.1
[25] tidyselect_1.2.0  glue_1.6.2        R6_2.5.1          rstatix_0.7.0
[29] fansi_1.0.3       pbdZMQ_0.3-8      carData_3.0-5     farver_2.1.1
[33] tidyr_1.2.1       ggplot2_3.3.6     purrr_0.3.5       car_3.1-0
[37] magrittr_2.0.3    backports_1.4.1   scales_1.2.0      htmltools_0.5.3
[41] assertthat_0.2.1  abind_1.4-5       colorspace_2.0-3  ggsignif_0.6.4
[45] labeling_0.4.2    utf8_1.2.2        munsell_0.5.0     broom_1.0.1
[49] crayon_1.5.2
```
